# Chronic muscle recordings reveal recovery of forelimb function in spinal injured female rats after cortical epidural stimulation combined with rehabilitation and chondroitinase ABC

**DOI:** 10.1002/jnr.25111

**Published:** 2022-08-02

**Authors:** Eleni Sinopoulou, Aline Barroso Spejo, Naomi Roopnarine, Emily R. Burnside, Katalin Bartus, Fred De Winter, Stephen B. McMahon, Elizabeth J. Bradbury

**Affiliations:** ^1^ Institute of Psychiatry, Psychology & Neuroscience King's College London, Regeneration Group, The Wolfson Centre for Age‐Related Diseases London UK; ^2^ Laboratory for Neuroregeneration Netherlands Institute for Neuroscience Amsterdam The Netherlands; ^3^ Department of Neuroscience, The Center for Neural Repair University of California San Diego California USA

**Keywords:** chondroitinase ABC, electrical stimulation, EMG, motor evoked potential, physical rehabilitation

## Abstract

Cervical level spinal cord injury (SCI) can severely impact upper limb muscle function, which is typically assessed in the clinic using electromyography (EMG). Here, we established novel preclinical methodology for EMG assessments of muscle function after SCI in awake freely moving animals. Adult female rats were implanted with EMG recording electrodes in bicep muscles and received bilateral cervical (C7) contusion injuries. Forelimb muscle activity was assessed by recording maximum voluntary contractions during a grip strength task and cortical motor evoked potentials in the biceps. We demonstrate that longitudinal recordings of muscle activity in the same animal are feasible over a chronic post‐injury time course and provide a sensitive method for revealing post‐injury changes in muscle activity. This methodology was utilized to investigate recovery of muscle function after a novel combination therapy. Cervical contused animals received intraspinal injections of a neuroplasticity‐promoting agent (lentiviral‐chondroitinase ABC) plus 11 weeks of cortical epidural electrical stimulation (3 h daily, 5 days/week) and behavioral rehabilitation (15 min daily, 5 days/week). Longitudinal monitoring of voluntary and evoked muscle activity revealed significantly increased muscle activity and upper limb dexterity with the combination treatment, compared to a single treatment or no treatment. Retrograde mapping of motor neurons innervating the biceps showed a predominant distribution across spinal segments C5–C8, indicating that treatment effects were likely due to neuroplastic changes in a mixture of intact and injured motor neurons. Thus, longitudinal assessments of muscle function after SCI correlate with skilled reach and grasp performance and reveal functional benefits of a novel combination therapy.

## Graphical abstract

Our work revealed that a combination treatment including ChABC, epidural cortical stimulation, and daily rehabilitation promotes increased muscle activity during grip strength while maximum voluntary contraction muscle recordings were acquired in awake behaving animals after a clinically relevant cervical contusion injury.

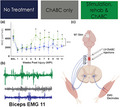

**Significance**

Chronic electrophysiological recordings have been used in the past to assess connectivity and conduction in spinal cord circuitry after injury, typically as a “snapshot” method or as terminal experiments. We longitudinally monitored biceps muscle activity in cervical contused rats from pre‐injury through to chronic post‐injury stages. This method detects physiological changes that correlate with skilled reach and grasp performance. We used this technique to assess the functional effects of a novel combination therapy (cortical epidural stimulation in conjunction with rehabilitative training and a neuroplasticity‐enhancing therapy). Our novel methodology revealed improved upper limb function after spinal cord injury and combination therapy.

## 1 INTRODUCTION

Traumatic spinal cord injury (SCI) affects more than 27 million people worldwide, with the global incidence expected to increase and currently no cure or no adequate therapies available (Ahuja et al., 2017; James et al., 2019). SCI therefore represents a significant global health burden for which new therapies are urgently needed. Despite some degree of spontaneous functional recovery that occurs following SCI (Fawcett et al., 2007; Steeves et al., 2011), most individuals with a SCI will experience permanent functional impairments and a lifetime of disability. Extensive physical rehabilitation is currently the most widely used approach to maximize any spontaneous recovery and improve functional outcome in the clinic (Field‐Fote, 2015; Harkema et al., 2012; Hicks et al., 2003), thus preclinical strategies for repair after SCI should be considered in the context of physical rehabilitation (Torres‐Espín et al., 2018). Experimental treatments that enhance neuroplasticity, for example, may be more efficacious when combined with neurorehabilitative strategies involving training and/or stimulation.

Chondroitinase ABC (ChABC) is a neuroplasticity‐inducing agent that degrades chondroitin sulfate glycosaminoglycan chains from chondroitin sulfate proteoglycan (CSPG) molecules, which are potent inhibitors of growth and neuroplasticity in the CNS (Burnside & Bradbury, 2014; Dyck & Karimi‐Abdolrezaee, 2015; Fawcett, 2015). Exogenous delivery of ChABC has been demonstrated to promote neuroplasticity, regeneration, and functional recovery in a number of SCI models across different species (Alilain et al., 2011; Bradbury & Carter, 2011; Bradbury et al., 2002; García‐Alías et al., 2009; Hu et al., 2018; Rosenzweig et al., 2019; Warren et al., 2018) and different levels of SCI (Bartus et al., 2014; James et al., 2015). Moreover, benefits of ChABC in the injured CNS can be augmented by rehabilitative interventions and recovery can be dependent on task‐specific activities (García‐Alías et al., 2009; Wang et al., 2011; Wiersma et al., 2017).

Another approach to improve functional recovery after SCI is by providing neuromodulation through cortical electrical stimulation (James et al., 2018). Evidence from studies involving noninvasive transcranial direct current stimulation (tDCS) and transcranial magnetic stimulation (rTMS) paradigms has demonstrated promising results in individuals with SCI. For example, when combined with extensive physical rehabilitation these paradigms can promote activity‐dependent induced plasticity (Sriraman et al., 2014) and improved upper limb function in skilled motor tasks (Gomes‐Osman & Field‐Fote, 2015). Furthermore, a clinical study in stroke sufferers demonstrated that more invasive subthreshold cortical epidural stimulation during extensive upper limb rehabilitation can lead to significant improvements and regain of motor function (Kleim et al., 2003), suggesting that this approach enables plasticity of motor pathways that may also prove beneficial for SCI. Accordingly, several preclinical studies have shown that extensive axonal sprouting of corticospinal projections can be achieved by daily epidural electrical stimulation of the motor cortex following pyramidotomy (Carmel et al., 2013, 2014), SCI (Jack et al., 2018; Zareen et al., 2017), and cortical lesions (Adkins et al., 2008) in adult rats. Additional experimental evidence from cortical ischemia studies has demonstrated that cortical electrical stimulation paired with rehabilitation leads to enhanced skilled motor recovery (Adkins‐Muir & Jones, 2013; Teskey et al., 2003). Thus, cortical electrical stimulation has been successfully used to target the injured CNS and it has promoted restoration of upper limb skilled function both in clinical and preclinical studies. Although electrical stimulation and rehabilitation paradigms are often applied in combination (Angeli et al., 2014; Capogrosso et al., 2018; Harkema et al., 2011; Sayenko et al., 2014) and neuroplasticity‐inducing agents have previously been combined with rehabilitation paradigms (Chen et al., 2017; García‐Alías et al., 2009; Wang et al., 2011), no studies to date have utilized a combination of a neuroplasticity‐promoting agent together with both cortical electrical stimulation and physical rehabilitation to promote recovery after SCI.

Here we aimed to assess a novel triple combination therapy in adult rats with cervical contusion injuries, an experimental model of the most common type of human SCI (Ahuja et al., 2017). We also aimed to apply tools available in the clinic to develop novel outcome measures of relevance to human SCI. A commonly used tool to assess muscle function after SCI in the clinic is electromyography (EMG) recordings, which can provide an indirect measure of muscle force and function, and significantly broaden the individual's clinical assessment (Curt et al., 1998). EMG recordings have been used in both preclinical experimental studies to examine the effects of CNS injuries in awake animals (Courtine et al., 2008; de Leon et al., 1998; García‐Alías et al., 2009; Xie et al., 2011) and in the clinic, by kinesiologists and physiotherapists to assess an individual's recovery of muscle function after a SCI (Calancie et al., 1994; Sherwood et al., 1996), and more recently assessed in a cervical SCI model up to 1 month post‐injury (Ganzer et al., 2016). Here, we established a novel longitudinal methodology of assessing muscle activity and function over a long‐term acute to chronic post‐injury time course after a cervical contusion injury by using chronic EMG forelimb recordings in awake, freely moving rats. We used this method to evaluate recovery of skilled hand function after treatment with a novel combination therapy, where the plasticity‐promoting therapeutic ChABC was combined with daily physical rehabilitation and cortical epidural stimulation. We hypothesized that, by utilizing the activity dependency of either spared or regenerating axons through extensive daily physical rehabilitation, in a ChABC‐induced permissive environment, electrical stimulation can augment activity in the injured spinal cord and aid their translation to skilled motor recovery. We demonstrate that using longitudinal EMG recordings post‐injury can provide sensitive physiological information about regain of muscle function and motor skills and show improved upper limb functional outcome with a novel combination therapy.

## 2 MATERIALS AND METHODS

### 2.1 Animals and experimental design

**FIGURE 1:**
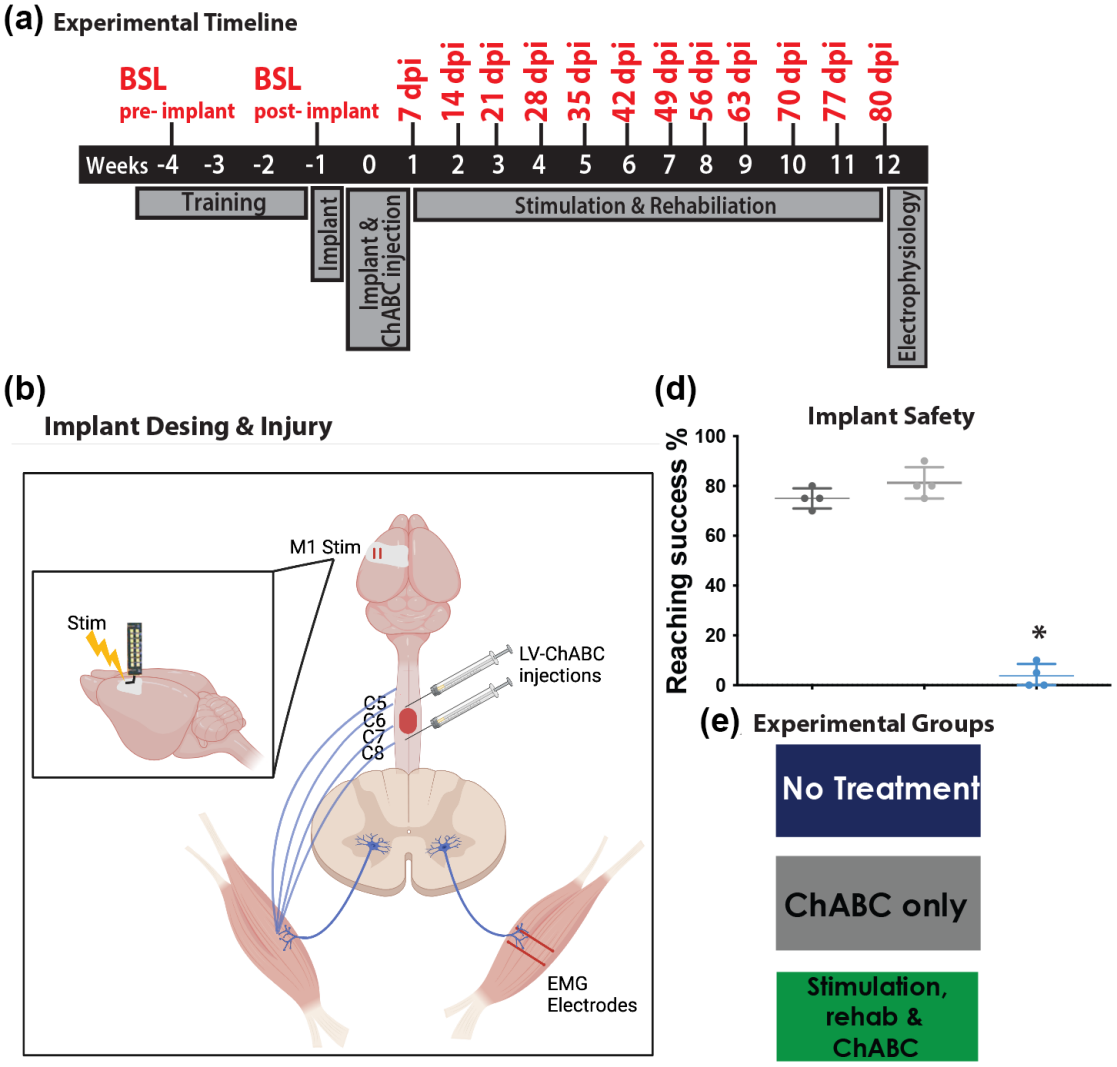
Experimental design. (a) Experimental timeline demonstrating experimental and surgical procedures (gray boxes) and experimental time points (in red). (b) Schematic shows a bilateral contusion injury at level C7 of the spinal cord (red circle), unilateral cortical epidural stimulation (M1 stim), EMG muscle recordings in the contralateral bicep muscle, and injections of LV‐ChABC rostral and caudal to injury. (c) Stimulating cortical epidural electrode over the motor cortex. (d) Whishaw window reaching success rate pre‐injury baseline (BSL) scores prior to implantation surgery (dark gray bar), and postimplantation surgery (light gray bar) and post‐contusion injury surgery (light blue bar) showing that implants do not affect the animals' performance in behavioral tasks (*F*_(3,12)_ = 1.700, *p* = .089 one‐way ANOVA), individual animals are plotted as symbols in the bar graph. (e) color‐coded experimental groups used for this study.

Adult female Lister Hooded rats (*n* = 42; 200–250 g; Charles River) were used for all experiments. We used only female rats in these studies because previous experience has shown that female Lister Hooded rats have a lower attrition rate for continuing with long‐term functional studies involving repeated testing than male rats of this strain. Female Lister Hooded rats also appear able to cope well with being single housed, with minimal or no signs of stress or aggression. Animals were single housed under a 12 h light/dark cycle with ad libitum access to food and water. Animal rooms were kept at 18–23°C (65–75 F) with a 45%–46% humidity and cages were changed once a week. All animals were handled for at least 2 weeks prior to the start of the study and were single housed after the initial surgery due to their implants. Due to single housing, all experimental animals received 15 min of supervised “playtime” (freedom to explore an enrichment arena, enriched with toys, textured materials, and tunnel tubes) five times a week. In‐cage enrichment was also provided in the form of chewable toys and paper. All procedures were performed in accordance with the United Kingdom Animals (Surgical Procedures) Act 1986 and approved by our institutional ethics committee (King's College London AWERB) and the U.K. Home Office (PPL PEE6F3C82). We first conducted a preliminary feasibility study in order to establish methodology for recording voluntary muscle activity in awake animals after SCI, to determine sensitivity of this methodology to effects of treatment, and to select treatment groups for subsequent longitudinal studies of muscle activity. Four experimental groups were assessed in the feasibility study, as follows: animals received spinal contusion injuries and either no treatment; cortical epidural stimulation only; cortical epidural stimulation plus daily behavioral rehabilitation; cortical epidural stimulation plus daily behavioral rehabilitation plus intraspinal LV‐ChABC injections (*n* = 4 for all groups). Assessments of biceps muscle activity were performed at a chronic (11‐week post‐injury) time point. This established feasibility of long‐term implantation of EMG electrodes and stable muscle recordings during a behavioral task. We subsequently conducted a longitudinal study, to evaluate temporal changes in voluntary and evoked muscle activity from acute to chronic injury (recording muscle activity weekly in the same animals via chronic implanted EMG electrodes). Three experimental groups were assessed in the longitudinal study, as follows: animals received spinal contusion injuries and either no treatment; intraspinal LV‐ChABC injection only; cortical epidural stimulation plus daily behavioral rehabilitation plus intraspinal LV‐ChABC injection (*n* = 6 for all groups) (Figure 1e). For this study, animals were assessed weekly for recovery of upper limb function using behavioral tasks (Whishaw window reaching and Montoya staircase) and muscle physiology (MEP recordings and voluntary muscle activity) over 11‐week post‐injury followed by terminal electrophysiology at week 12. Animals were randomly assigned in the different treatment groups and received 4 weeks of behavioral training prior to their electrode implantation surgeries, followed by contusion injuries 1 week later (Figure 1 shows the experimental design and timelines for the longitudinal muscle activity study).

### 2.2 Electrode manufacturing

An epidural bipolar tungsten stimulating electrode with a length of 2 mm was used for stimulating the motor cortex (M1) over the caudal forelimb area (MS303T/2; P1 technologies, Inc). The tips of the electrode were de‐insulated for better dura contact using a Dremel. EMG electrodes were constructed from a 7‐stranded Teflon coated stainless steel wire (A‐M Systems, #793500). At the end of the electrode wire, a 25G hypodermic needle was crimped for easy insertion (perpendicular) intramuscularly, which was then cut off post‐insertion. These electrodes had approximately 2 mm of de‐insulated wire near the end, which was placed inside the muscle to record muscle activity. To ensure the quality of the EMG electrodes both their continuity and impedance were measured before implantation. The impedance for all the EMG electrodes was between 7 and 12 kΩ. For recording EMG signals an interface board (Electrode Interface Board; EIB‐16, Neuralynx) was used, utilizing a 16‐channel connector (Omnetics). Each EMG recording channel was paired to record differentially from the respective muscles. This allowed for reduced electrical noise in the signal. Wires from the periphery passed subcutaneously and were attached to the interface board using gold plated pins. All EMG electrodes were referenced with a wire connected to a skull screw placed on the animal's head cap.

### 2.3 Surgical procedures

#### 2.3.1 Primary motor cortex (M1) epidural electrode implantation

The rats were anesthetized (ketamine, 60 mg/kg, and medetomidine, 0.25 mg/kg) and the back of the neck and both forelimbs were shaved and cleaned with iodine. Core temperature was maintained close to 37°C using a self‐regulating heated blanket for the duration of the procedure. The electrode was sterilized with 10% Clorox and then rinsed with 70% isopropyl alcohol and sterile saline. The head was fixed in a stereotaxic frame and a small craniotomy was drilled at the level of bregma, extending from 1 to 3 mm lateral to midline, and from 1 to 3 mm rostral to midline. After the craniotomy on the M1 site, the electrode was placed on the dura over the caudal forelimb area, 1 mm lateral to midline and 1 mm caudal to bregma (Figure 1c), contralaterally to the dominant forepaw, as previously determined by single pellet reaching. The electrode/craniotomy site was covered with gel foam to avoid dental acrylic contact with the dura. Finally, the electrode was stabilized with dental acrylic bridged to skull screws.

#### 2.3.2 EMG electrode implantation

The EMG electrodes were sterilized with 10% Clorox and then rinsed with 70% isopropyl alcohol and sterile saline. They were then tunneled under the skin from the scalp incision site and were inserted 2–4 mm apart in the belly of the *biceps brachii* muscle group (Figure 1b). The bicep muscle was chosen due to its larger size and its involvement in the skilled reaching task. The EIB was placed on the skull and stabilized with dental acrylic along with the epidural stimulating electrode creating a secure head cap. In all animals, a loop was made with the extra length of the EMG electrode wire near the nape of the neck to allow for unrestricted limb and head movement. After the electrode implantation procedure, all animals received postoperative analgesia (carprofen 5 mg/kg) for 2 days, saline for rehydration (3–5 ml) for 3 days, and a 1 week course of antibiotics (Baytril; 5 mg/kg).

#### 2.3.3 Contusion SCI

One week after electrode implantation all animals underwent cervical contusion injuries, as previously described (Burnside et al., 2018; James et al., 2015). Rats were anesthetized (ketamine, 60 mg/kg, and medetomidine, 0.25 mg/kg), the cervical region of their backs was shaved and cleaned with iodine and core temperature was maintained close to 37°C using a self‐regulating heated blanket. A laminectomy was performed at vertebral levels C5 and C6 to expose the underlying spinal cord. Animals were then positioned and stabilized in an Infinite Horizon impactor (Precision Systems and Instrumentation, Lexington, KY) with the impactor probe positioned 3–4 mm above the exposed spinal cord. The impactor probe delivered an impact force of 225 kD to the C7 spinal cord, inducing a moderate midline cervical contusion injury. Following the injury, rats designated to a treatment group that did not include LV‐ChABC intraspinal injections had their wounds sutured and anesthesia was then reversed using atipamezole hydrochloride (1 mg/kg administered i.p.). In the acute postoperative period following the contusion injury, all animals received extensive welfare checks and during this period animals were housed in a recovery holding incubator for at least 48 h in absorbent lined cages with soft bedding and less sawdust, to maximize ability to locomote, and were provided with hydration gel and modified diet before returning to standard husbandry conditions. Following contusion surgery, all animals received postoperative analgesia (carprofen 5 mg/kg) for 5 days, saline for rehydration (3–5 ml), and a 1 week course of antibiotics (Baytril; 5 mg/kg).

#### 2.3.4 Chondroitinase gene, lentiviral vector (LV‐ChABC), and intraspinal injections

The *Proteus vulgaris* ChABC gene was modified with mutations targeted to remove five cryptic *N*‐glycosylation sites and addition of a mammalian signal sequence and resynthesized with mammalian preferred codons to make a mammalian‐compatible engineered ChABC gene (Muir et al., 2010). The modified ChABC cDNA was subcloned into a lentiviral transfer vector (termed LV‐ChABC) with the mouse phosphoglycerate kinase (PGK) promoter, as previously described in detail (Bartus et al., 2014). The resulting vector was integrated, self‐inactivated, and pseudotyped with VSV‐G (vesicular stomatitis virus G). Viral particles were concentrated by ultracentrifugation and titered by qPCR as described previously (Burnside et al., 2018). The resulted viral titer used for both studies was 1.03E10 GC/ml (gift from Joost Verhaagen, Netherlands Institute for Neuroscience). The animals designated to receive LV‐ChABC treatment received two 0.5 μl intraspinal injections of LV‐ChABC immediately after the injury, rostral, and caudal to the contusion site (one injection per side). Injections were delivered at a rate of 200 nl/min using an Ultramicro Pump III (World Precisions Instruments, Europe). Following the intraspinal injection, wounds were sutured, and anesthesia was reversed. All animals received extensive postoperative care, as described above.

### 2.4 Behavioral rehabilitation and assessment

The Whishaw window reaching and the Montoya staircase tasks were utilized for daily behavioral rehabilitation and to assess recovery of upper limb function. All rats were habituated and trained on the tasks for 4 weeks prior to any surgical procedures and baseline scores were obtained prior to electrode implantation and prior to contusion surgery. For the Whishaw window reaching task, the animals were trained to repeatedly reach for sugar pellets (Test Diet, Sandown Scientific) through a slit in a Plexiglas cage. For behavioral rehabilitation, animals performed the task daily (5 days/week) for 15 min starting at 1 week post‐injury. During the weekly behavioral assessment animals received a total of 20 pellets and were scored on how many they successfully retrieved and ate. For the Montoya staircase task, animals were placed in a chamber leading to a platform with access to a welled staircase on both sides where animals were trained to bilaterally reach for the sugar pellets placed in the wells. As with the reaching task, for the behavioral rehabilitation animals performed this task daily (5 days/week) for 15 min starting at 1 week post‐injury. During the weekly behavioral assessment animals were scored on how many out of a total of 28 (14 per side) pellets were successfully retrieved and eaten. Colored pellets were used to identify the levels of success and pellet displacement in this task.

### 2.5 Epidural cortical stimulation

Epidural cortical stimulation started 1 week post‐injury after animals had recovered from the acute postoperative stages following contusion injury surgeries. The animals were transferred from their home‐cage to a stimulating chamber and acclimatized for 10 min before every session. Cortical epidural stimulation was administered immediately after the daily rehabilitation protocol was concluded. The cortical stimulation protocol consisted of subthreshold epidural stimulation of the motor cortex via epidural implanted electrodes for 3 h daily for 5 days/week (five pulses at 500 Hz, 0.2 ms biphasic pulses, every 2 s) for a total of 10 weeks (52 days) for all animals designated to a treatment group that included epidural stimulation. Our paradigm was based on previous studies by the Martin lab (Brus‐Ramer et al., 2007; Carmel et al., 2010, 2013, 2014) and our parameters were adjusted from Meng et al. (2004), where spinal cord postsynaptic potentials were evoked using similar parameters. These previous studies applied 6 h of subthreshold cortical electrical stimulation daily for 10 consecutive days at a 333 Hz. We used a modified paradigm, stimulating for a shorter period of time daily while using a more prolonged paradigm over time. Evidence from previous studies demonstrated that 10 days of cortical electrical stimulation can elicit improvements in skilled function in tasks such the horizontal ladder, with effects lasting up to 30 days after completion of the stimulation paradigm (Li et al., 2010). We hypothesized that we could achieve a more robust and long‐lasting effect with a longer stimulation paradigm, continuing over the entire 11 week study period. We also used a higher frequency than previous studies, hypothesizing that we could harvest a similarly lasting effect.

### 2.6 Chronic electrophysiological assessments and analysis

#### 2.6.1 Motor evoked potential (MEP) recordings

Evoked muscle responses were assessed by stimulating at threshold intensity and at 1.5 times the threshold intensity. To achieve this, animals were connected to a recording acquisition system (Neuralynx; CED), and to an isolated stimulation unit (A‐M Systems). The threshold value in milliamps (mA) was defined as the minimal amount of current needed to produce an evoked overt bicep contraction. The muscle activity evoked by the cortical epidural electrical stimulation was recorded and 30 triggers at a sampling rate of 20 kHz were acquired (100–300 kHz bandwidth) using Signal software from a commercial system (CED Power 1401, SIGNAL; Cambridge Electronic Design Ltd). Muscle activity was recorded from the biceps muscle group. Similar to the 3‐h stimulation period, during MEP recordings animals were also awake, unrestrained and able to freely move in the chamber. There were three aspects that helped reduce signal noise and variability due to movement artifacts: (i) by using differential recording electrodes, (ii) by recording 60 triggers and then manually selecting 20, enabling the selection of triggers with minimal spontaneous EMG activity (more details in *Analysis of electrophysiological recordings* section) and (iii) by averaging these 20 selected triggers, which enables a robust response by canceling spontaneous EMG activity from movements.

#### 2.6.2 Maximum voluntary contraction (MVC) recordings

Voluntary muscle responses were assessed by measuring the animals' MVC during a forelimb grip strength task. The trial started as the animal was placed on the grip strength apparatus platform and lasted for approximately 8 s. Rats grasped onto a metal bar of a forelimb grip strength meter (Linton Instrumentation) with their forepaws and were slowly pulled back until they were unable to hold on and would then release the bar. As previously described (Aguilar et al., 2010; James et al., 2015), a trial was counted as successful when the animal managed to grasp and hold on to the metal bar by flexing its digits and then releasing the bar by extending its digits. The amount of muscle activity exerted by the bicep muscle group during the pull was recorded, timestamped, and measured. The animals performed the task three times and the time interval between each trial was <15 s. The averaged values from the rectified traces were calculated. During this task the grip strength force measured for each forelimb by the grip strength apparatus was also recorded. The same experimenter carried out the test throughout the entire duration of the study to ensure consistency of testing parameters.

#### 2.6.3 Terminal electrophysiology experiments

Terminal experiments were performed following the last behavioral time point at 12 weeks post‐injury (in *n* = 4 per group). Animals were anesthetized (ketamine, 60 mg/kg, and medetomidine, 0.25 mg/kg) and the depth of anesthesia was regularly assessed by monitoring pedal withdrawal reflexes and respiratory rate. Core temperature was maintained close to 37°C using a self‐regulating heated blanket. The experiment consisted of two stages. The first stage involved stimulating through the implanted cortical electrode at maximum current (10 mA, 2 pulses, 0.2 ms biphasic pulses, every 30 s) and recording from percutaneously placed EMG wires, similarly to the awake stimulating MEP protocol. The second stage consisted of using a needle electrode to stimulate the implanted EMG electrode in the bicep muscle at threshold intensity (single pulses at 500 Hz, 0.2 ms biphasic balanced pulse, every 2 s) through the appropriate channel in the EIB interface. During both stimulation paradigms the muscle activity was recorded through a pair of percutaneous EMG wires placed on the bicep muscle for differential recordings. The percutaneous EMGs were manufactured in‐house using a 27‐gauge needle with a hooked wire on the tip and they were referenced to a ground wire on the back of the animal.

#### 2.6.4 Analysis of electrophysiological recordings

To analyze the MEP recordings, EMG waveforms were first rectified and then filtered through a 5‐point smoothing algorithm, a low pass filter (230 Hz, 310 decibels) and the DC offset was corrected. Moreover, to ensure no movement artifacts would be included in the averaging, a total of 20 clean triggers were manually chosen from the 60 acquired and were then averaged. The area under the curve (AUC) of the EMG average was measured for each animal at each threshold intensity and at each time point. The basic criteria to recognize the MEP response were a constant 15% deviation of the signal from the baseline for onset and offset of response. Data from terminal electrophysiology experiments underwent the same analysis. To analyze the MVC recordings, raw traces were filtered through a low pass filter (230 Hz, 310 decibels) and then the timestamped portions were rectified. To get the final AUC value, three trials per animal were measured and averaged. MVC muscle activity was quantified by averaging AUC values recorded from both forelimbs.

### 2.7 Tissue processing, histology, and imaging

At the study end point (12 weeks post‐injury), animals were deeply anesthetized with sodium pentobarbital (Euthatal, 80 mg/kg, i.p.) and transcardially perfused with 0.9% saline followed by 4% paraformaldehyde in 0.1 M phosphate buffer. Immediately after perfusion, spinal cord tissue was dissected (~10 mm with the lesion epicenter located centrally for lesion spread and chondroitin‐4‐sulfate (C‐4‐S) assessments; from C2 to T2 for motor neuron mapping assessments), postfixed overnight in 4% PFA, washed in PBS and kept in sucrose 30% for 5 to 7 days, then embedded and frozen in Optimum Cutting Temperature Compound before being cut into serial (20 μm) sections, either cut transversely (for C‐4‐S and EC) or horizontally (for biceps motor neuron mapping).

#### 2.7.1 C‐4‐S staining and imaging

To confirm successful intraspinal delivery of LV‐ChABC, sections spanning the injury epicenter were stained for C‐4‐S using tyramide signal amplification, to reveal stub epitopes which are only present after degradation of chondroitin sulfate glycosaminoglycans (CS‐GAGs). Sections were rehydrated with PBS and incubated at room temperature in the following: hydrogen peroxide (0.3%, 20 min), mouse monoclonal anti‐C‐4‐S (MP Biomedicals; 1:5000, overnight), biotinylated secondary antibody (anti‐mouse biotin; Vector Laboratories, USA; 1:400, 2 h), ABC reagent (ThermoFisher Scientific, USA; 1:250, 30 min), biotinyl tyramide (PerkinElmer Life Sciences, Boston, MA; 1:75, 10 min), and extra‐avidin FITC (Sigma‐Aldrich; 1:500, 3 h). Slides were coverslipped using Fluoromount mounting medium with DAPI (#CO‐4959‐52, Invitrogen). Representative images of C‐4‐S immunostaining for each treatment group were obtained using a confocal microscope (Zeiss, LSM710), using identical exposure and settings.

#### 2.7.2 Eriochrome cyanine (EC) histochemistry

To identify the lesion epicenter and determine the extent of lesion spread to other spinal levels, transverse sections of the spinal cord spanning the lesion were histochemically stained with EC. Slides were dehydrated in graded ethanol series followed by clearing with xylene and subsequently rehydrated in reverse ethanol series followed by distilled water. Slides were stained in EC 0.16% (with 0.5% sulfuric acid and 0.4% iron chloride in distilled water) for 10 min, followed by distilled water washes and differentiation in 0.5% aqueous ammonium hydroxide for 35 s. This was followed by washes in distilled water and dehydration in graded ethanol series followed by clearing with xylene. Sections were mounted in entellan (1.07960.0500, Sigma‐Aldrich) and coverslipped. Images were acquired using an Axioplan 2 imaging microscope (Zeiss).

#### 2.7.3 Biceps motor neuronal pool mapping

To map the segmental distribution of spinal motor neurons innervating the *biceps brachii* in naïve (uninjured) and SCI rats, the retrograde tracer Cholera Toxin B Subunit (CTB) was intramuscularly injected, and traced motor neurons were counted at each spinal level from C2 to T2.

#### 2.7.4 Tracer intramuscular injection

Adult female Lister Hooded rats (220–290 g; Charles River; husbandry conditions as above), either naïve (*n* = 5) or 10 weeks after SCI (spinal C7 bilateral contusion, as above; *n* = 3) had the right *biceps brachii* intramuscularly injected with CTB. Rats were anesthetized with isoflurane (5% induction and 2.5% maintenance in 1 L/min O_2_). Analgesia (carprofen, CarprieveTM, 5 mg/kg) was administered subcutaneously, and the right forelimb was shaved and disinfected with 4% w/v chlorhexidine gluconate. The skin was incised and retracted, and the fascia was carefully opened for clear visualization of the two heads of the muscle. A total of 12 μl of 0.5% CTB diluted in distilled water (104 Cholera Toxin B Subunit; #10433A1, List Biological Laboratories, Inc.) was injected into the right biceps using 10 μl Hamilton syringes (#80300, 26s‐gauge, Hamilton). The short head of the biceps received 8 μl of CTB, divided into two deep injections of 2 μl and two superficial injections of 1 μl aiming the motor endplates (Tosolini & Morris, 2012) and two deep injections of 1 μl aiming at the region halfway between the proximal end of the muscle and the motor endplates (Figure 6a). The long head of the biceps received 4 μl of CTB, divided into one deep injection of 2 μl and one superficial injection of 1 μl aiming the motor endplates, and one deep injection of 1 μl aiming the region halfway between the proximal end of the muscle and the motor endplates (Figure 6a). The muscle was wiped before and after injections with a cotton bud. The needle was inserted parallel to the muscle fibers, the tracer was injected slowly, and the needle was kept in place after injection for at least 30 s. After injections, the skin was sutured, and saline administered subcutaneously. Rats recovered from anesthesia in a heated recovery chamber.

#### 2.7.5 Tissue preparation

Seven days after CTB tracer injection, all rats were deeply anesthetized and transcardially perfused as above (other than one animal in the SCI injury group, which was perfused at 4 days after tracer injections for welfare reasons; SCI rat #1). The spinal cord with DRGs attached from the second cervical level to the second thoracic level was dissected, postfixed and cryopreserved as above. Fiducial marks (needle pin) were made to determine the two limits of spinal levels C4 and C8. The limits of the spinal levels were determined halfway between the upper and lower roots (Tosolini & Morris, 2012), and spinal levels were identified according to their anatomical position in relation to the vertebrae (Harrison et al., 2013) and confirmed by DRG counting. DRGs were removed after fiducial marking, and spinal cords were embedded in OCT and frozen as above. Horizontal sections of the spinal cord at a thickness of 20 μm were obtained using a cryostat. Slides were stored at −20°C.

#### 2.7.6 CTB staining and imaging

Every other section of the spinal cord (20 μm apart) was immunostained for CTB. The slides were washed with PBS, blocked for 1 h in 3% BSA in PBS (Bovine Serum Albumin; #A3059; Sigma‐Aldrich) and incubated overnight in the primary antibody goat anti‐CTB (#7032A10; List Biological Laboratories, Inc.) 1:4000 in 0.2% Triton‐x100 and 1% BSA in PBS. Slides were then washed in PBS and incubated with the secondary antibody Alexa Fluor 488 (#A11055; Invitrogen) 1:1000 in 0.2% Triton‐x100 and 1% BSA in PBS for 3.5 h protected from light. After PBS washes, the slides were coverslipped using Fluoromount mounting medium with DAPI (#CO‐4959‐52, Invitrogen). All sections with positive motor neurons were imaged using a VS120 Olympus slide scanner using DAPI and FITC filters to visualize cell nuclei and CTB, respectively. The software QuPath was used to draw individual spinal levels and count neurons in each spinal level. The presence of the fiducial marks determined the limits of C4 and C8 levels, and the other levels were determined by distance. For each animal, the total number of CTB positive neurons present in each spinal level (from C2 to T2) was calculated.

### 2.8 Statistical analysis

GraphPad prism 9.2.0 was used to perform statistical analyses. For single time point recorded data, statistical analysis was performed using one‐way ANOVA with supplementary Tukey's and/or Bonferroni's *post‐hoc* tests. For all chronically acquired data, a two‐way repeated measures ANOVA was performed, with supplementary Dunnett's, Tukey's, or Bonferroni's *post‐hoc* tests. All data are presented as mean ± standard deviation (SD) values. For motor neuron mapping data, comparing naïve and SCI groups, multiple Mann–Whitney tests (Mann–Whitney *U* test) were performed. Differences were considered significant at *p < .05, **p < .01, and ***p < .001. For all two‐way repeated measures ANOVA statistical analyses, a complete data set was used without any missing values and/or time points. Furthermore, normal distributions of the dependent variables and equal variances between experimental groups were obtained.

### 2.9 Blinding and randomization

During surgical procedures the experimenter who performed the intraspinal injections was unaware of the animals' identification numbers and to which treatment group each animal was assigned. All behavioral assessments were recorded on camera during the task and were then analyzed by an experimenter blinded to treatment. The chronic electrophysiology acquired data were analyzed after the file names were changed to blind the experimenter to treatment group. Finally, the experimenter performing the electrophysiological terminal experiments was also blinded to each animal's treatment group. Therefore, blinding was maintained as far as possible during data collection and analysis.

## 3 RESULTS

### 3.1 Feasibility study: Endpoint MVC muscle recordings

A feasibility study was first conducted to evaluate the methodology for recording voluntary muscle activity in awake animals during a forelimb grip strength behavioral task. This study also determined the choice of treatment groups for chronic longitudinal electrophysiological assessments. MVC is a traditional way of testing the maximum amount of force that can be voluntarily exerted from a muscle (Thomas et al., 1997). The grip strength apparatus was used for the rats to perform this task while connected to the recording interface at 11 weeks post‐injury. MVC recordings, measured as AUC, revealed that the triple combination treatment group showed a significant increase (*F*_(3,12)_ = 12.86, *p* < .001 one‐way ANOVA, Tukey's *post‐hoc*) compared to the group receiving stimulation only and the untreated group (Figure S1a,b), suggesting that animals which received the triple treatment combination had a significant increase in voluntarily exerted muscle activity. The stimulation and rehab group also appeared to show an increase in MVC, although they were not significantly different from either the stimulation only and untreated groups, or the triple treatment combination group. Interestingly, when the force values produced by the grip strength apparatus were investigated, this did not reveal any significant differences between groups (*F*_(3,12)_ = 1.700, *p* = .089 one‐way ANOVA) (Figure S1c), indicating that recording MVC muscle activity may be a more sensitive method for revealing changes in muscle function after contusion injury in rats than the grip strength behavioral task alone. Based on the feasibility study, we selected the most promising treatment paradigm for subsequent longitudinal studies, to comprehensively assess changes in both voluntary and evoked muscle activity over time in the same animals. Thus, the triple combination treatment paradigm was selected, alongside a no treatment group. A LV‐ChABC single treatment group was also added as a comparison, since other single and double treatment paradigms showed only modest or no effects in the feasibility study.

### 3.2 Longitudinal study: MVC muscle recordings over acute to chronic post‐injury time points reveal increased voluntary muscle activity in animals treated with a triple treatment combination

**FIGURE 2:**
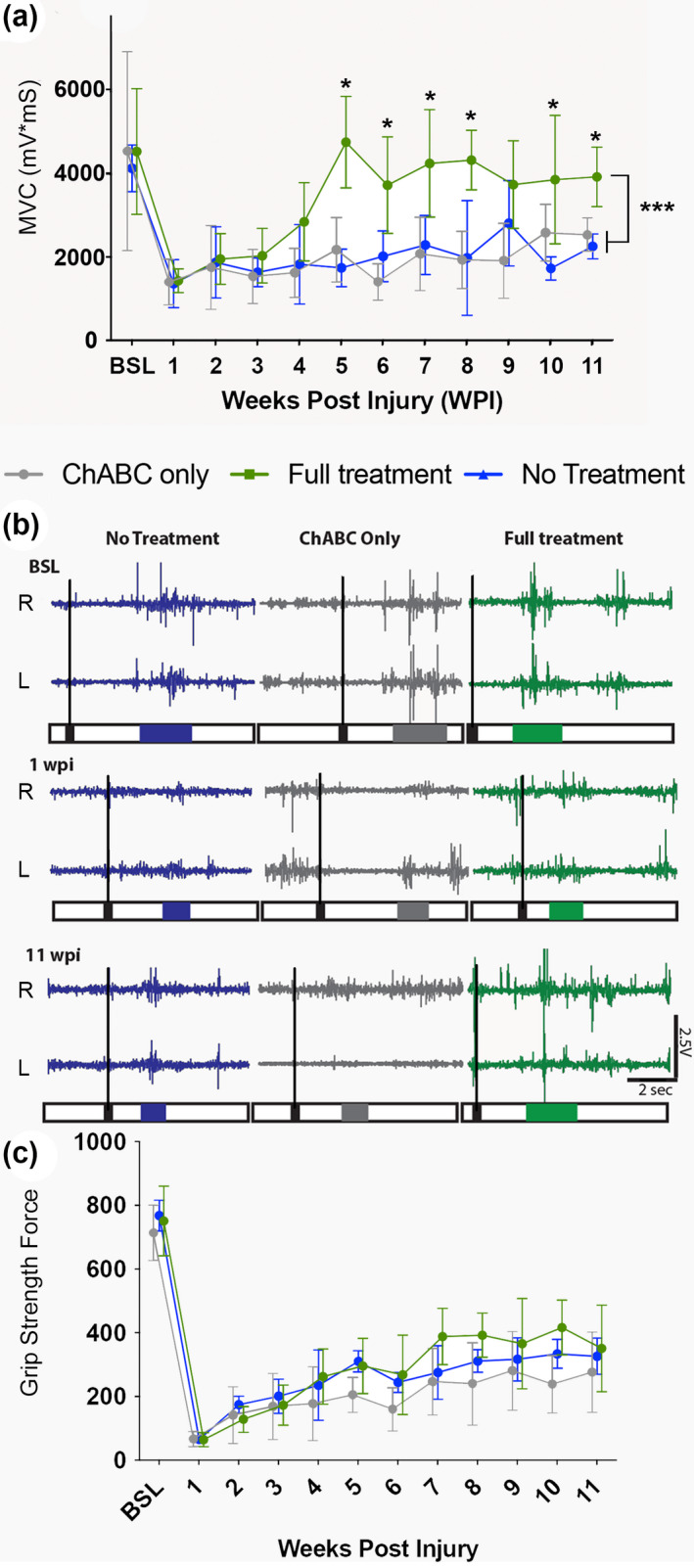
A triple combination treatment promotes increased muscle activity during voluntary behavior. (a) Weekly maximum voluntary contraction (MVC) recordings measured in awake animals during a behavioral (grip strength) task reveals a significant increase in MVC in the triple treatment group, which begins to emerge at 4 weeks post‐injury and is significantly increased compared to the untreated or LV‐ChABC only treatment groups at all subsequent time points (Time: *F*_(4.793,71.90)_ = 15.96, *p* < .0001; Treatment: *F*_(2,15)_ = 16.08, *p* < .001; Interaction: *F*_(22,165)_ = 3.075, *p* < .0001, two‐way RM ANOVA, Dunnet's *post‐hoc*). (b) Raw traces during MVC recordings for right (R) and left (L) forelimbs at three time points (BSL, 1 week post‐injury and 11 weeks post‐injury) recorded from the same animal over time, averages of three trials. Colored traces indicate the different groups, outlined boxes indicate the response duration, and black boxes indicate the timestamped start of each trial. (c) In contrast to the electrophysiological evaluations, weekly grip strength behavioral testing revealed no differences between groups in the force measurement values evaluated over the 11 week time course (Time: *F*_(11,55)_ = 90.77, *p* < .0001, Treatment: *F*_(2,10)_ = 5.276, *p* < .05; Interaction: *F*_(22,110)_ = 1.789, *p* < .05 two‐way RM ANOVA, Bonferroni's *post‐hoc*).

After establishing a robust outcome measure for assessing voluntary muscle activity in awake behaving animals at a single chronic time point post‐injury, and initial assessments with single, double, and triple treatment combinations, we then applied this methodology to a longitudinal 11 week study, with weekly physiological and behavioral assessments to get a fuller picture of recovery following the triple combination treatment therapy. Here, we compare the triple combination treatment group (cortical epidural stimulation plus daily behavioral rehabilitation plus intraspinal LV‐ChABC injection) with a group that received LV‐ChABC treatment alone and an untreated group. Long‐term muscle activity recordings revealed that the group which received the triple combination of treatments showed a significant increase in muscle activity, when compared both to the LV‐ChABC alone group and the untreated group (Time: *F*_(4.793,71.90)_ = 15.96, *p* < .0001; Treatment: *F*_(2,15)_ = 16.08, *p* < .001; Interaction: *F*_(22,165)_ = 3.075, *p* < .0001, two‐way RM ANOVA, Dunnet's *post‐hoc*) (Figure 2a). BSL muscle activity recordings for all groups showed a similar muscle activity range prior to cervical contusion injury and a decrease in the activity was observed, as expected, at 1 week post‐injury. Starting at 4 weeks post‐injury the triple combination treatment group showed an increase in muscle activity, which reached a plateau after 6 weeks post‐injury and was maintained until the end of the study. These results suggest a lasting increase in connectivity in spinal cord circuitry, where the treatment combination may act to restore damaged circuitry and/or enable re‐routing of spared rostral circuitry, which enables some recovery of upper limb function after injury. This is also indicated by the raw traces (Figure 2b). Interestingly, as observed in the feasibility study, the force values recorded from the grip strength apparatus did not reveal a difference between the groups over time (Time: *F*_(11,55)_ = 90.77, *p* < .0001; Treatment: *F*_(2,10)_ = 5.276, *p* < .05 Interaction: *F*_(22,110)_ = 1.789, *p* < .05, two‐way RM ANOVA, Bonferroni's *post‐hoc*) (Figure 2c). The lack of correlation with improved bicep muscle activity and grip strength behavior likely reflects heterogeneous muscle engagement during grip strength, rather than being exclusive to the biceps. Thus, we provide evidence that recording MVC muscle activity provides a sensitive method for revealing changes in muscle function over time after contusion injury.

### 3.3 Chronic muscle recordings reveal increased evoked muscle activity in animals treated with a triple treatment combination

**FIGURE 3:**
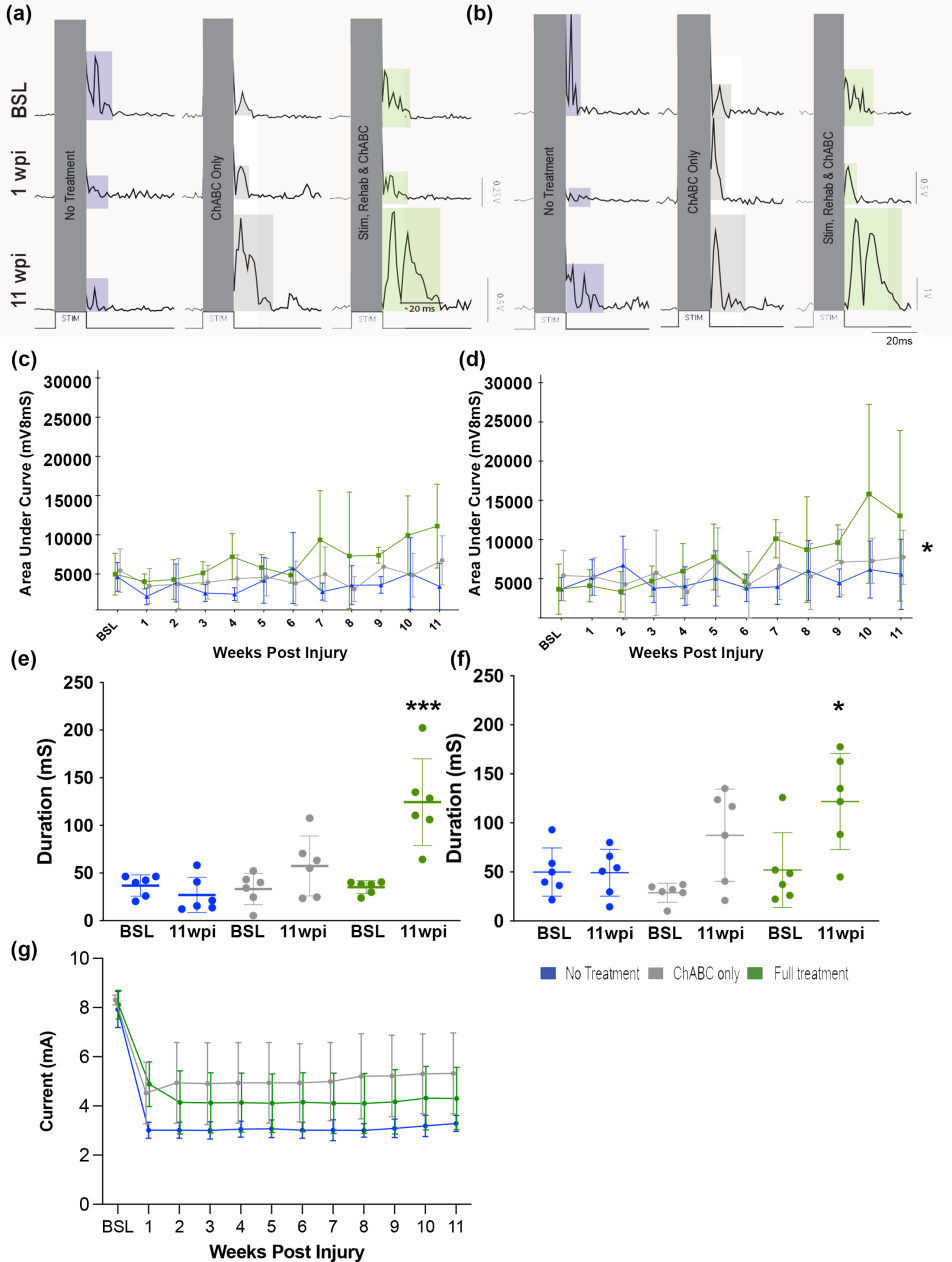
A triple combination treatment promotes increased muscle activity during evoked cortical stimulation. Weekly motor evoked potential (MEP) recordings acquired in awake freely moving animals. Rectified average traces (15 triggers average) of MEPs from the different groups at three different time points (BSL, 1 week post‐injury and 11 weeks post‐injury) at (a) threshold intensity and at (b) 1.5 times the threshold intensity recorded from the same animal over the time course of 11 weeks. Note different *y*‐axis scale bar for 11 weeks post‐injury at threshold values to accommodate for better trace representation. Quantified MEP weekly recordings, at (c) threshold intensity and at (d) 1.5 times the threshold intensity. The triple treatment group showed a significant increase in MEPs when compared to the no treatment or LV‐ChABC only treatment groups (Time: *F*_(11,55)_ = 3.422, *p* = .001; Treatment: *F*_(2,10)_ = 7.809, *p* < .01; Interaction: *F*_(22,110)_ = 1.679, *p* < .05 two‐way RM ANOVA, Bonferroni's *post‐hoc*) when stimulated at higher current intensity (d). Duration of MEP AUC response compared between the treatment groups at BSL and at 11 weeks post‐injury at (e) threshold intensity and at (f) 1.5 times the threshold intensity. The triple treatment group at 11 weeks post‐injury showed responses with significantly longer duration than the no treatment or LV‐ChABC only treatment groups (e, *F*_(5,30)_ = 1.978, *p* < .0001, one‐way ANOVA, Bonferroni's *post‐hoc*; f, *F*_(5,30)_ = 1.822, *p* < .0001, one‐way ANOVA, Bonferroni's *post‐hoc*;). (g) Current (mA) threshold intensities used for therapeutic stimulation remained stable throughout the experiment (Time: *F*_(2,45)_ = 73.79, *p* < .0001; Treatment: *F*_(2,45)_ = 12.98, *p* < .001; Interaction: *F*_(4,45)_ = 2.266, *p* = .0768, two‐way RM ANOVA, Tukey's *post‐hoc*).

MEPs were recorded from bicep contraction and forelimb flexion, in awake, freely moving animals, evoked by epidural cortical electrical stimulation. First, we assessed whether the EMG electrode implantation could affect the outcome of behavioral tasks. After obtaining presurgery baselines, all animals were reevaluated 1 week post‐electrode implantation surgery on the Whishaw window reaching task and the scores for successfully reached pellets postimplantation were not different from those of the prior‐implantation baselines (Figure 1d). However, as expected, the reaching success was significantly depleted after the animals received a cervical contusion injury (*F*_(2,69)_ = 544, *p* < .001 one‐way ANOVA, Bonferroni's *post‐hoc*) (Figure 1d). Therefore, implantation procedures did not have an adverse effect on the animals' behavioral performance. MEPs were recorded at threshold value (Figure 3a,c,e) over an 11 week period from the bicep muscles contralaterally to the stimulating electrode. While a statistically significant change was not observed in the AUC measurements, there was a trend suggesting that the triple treatment group exhibits an increase in MEPs after injury (Figure 3c). When MEPs were recorded at 1.5 times the current threshold intensity (Figure 3b,d,f), a statistically significant increase in AUC (Time: *F*_(11,55)_ = 3.422, *p* < .001; Treatment: *F*_(2,10)_ = 7.809, *p* < .01 Interaction: *F*_(22,110)_ = 1.679, *p* < .05, two‐way RM ANOVA, Bonferroni's *post‐hoc*) was observed in the triple treatment combination group when compared to the LV‐ChABC only and untreated groups (Figure 3d). This suggests that the use of higher current intensity can reveal activity changes, due to a larger amount of muscle fibers being recruited and a more effective signal conduction occurring below injury. We also demonstrate these results in the rectified example traces (Figure 3a,b) for both threshold intensities. Finally, to assess the overall effect of the treatment combination on muscle activity, the duration of the MEP response was measured and quantified at BSL and at 11 weeks post‐injury for threshold stimulation intensity (Figure 3e) and 1.5 times the threshold stimulation intensity (Figure 3f). As expected, the duration of the response observed during BSL recordings, in both current intensities, was not significantly different between the experimental groups. However, at 11 weeks post‐injury the group that received the triple treatment combination showed a significant increase in MEP response duration compared to both the LV‐ChABC only and the untreated groups when stimulated at threshold intensity (*F*_(5,30)_ = 1.978, *p* < .0001, one‐way ANOVA, Bonferroni's *post‐hoc*; Figure 3e), and compared to the untreated group when stimulated at 1.5 times threshold intensity (*F*_(5,30)_ = 1.822, *p* < .0001, one‐way ANOVA, Bonferroni's *post‐hoc*; Figure 3f). Finally, we confirmed that the stimulation threshold values for evoking MEP responses were stable and reproducible throughout the 11‐week stimulation study (Figure 3g), thus further demonstrating the stability of our longitudinal recording technique. Furthermore, we performed a group comparison which revealed no statistically significant differences between our treatment and no treatment groups (Time: *F*_(2,45)_ = 73.79, *p* < .0001; Treatment: *F*_(2,45)_ = 12.98, *p* < .001; Interaction: *F*_(4,45)_ = 2.266, *p* = .0768, two‐way RM ANOVA, Tukey's *post‐hoc*). These results reveal that epidural stimulation of the motor cortex evokes a response which can be robustly recorded over the course of 12 weeks using our novel methodology. Furthermore, investigating the effects of stimulation in combination with other therapeutic treatments shows that the triple combination of treatments leads to increased muscle activity and response duration when compared to a single LV‐ChABC treatment or no treatment, and suggests there may be some recovered spinal cord circuitry due to the treatment combination.

### 3.4 Increased muscle activity is associated with regain of skilled forelimb function

**FIGURE 4:**
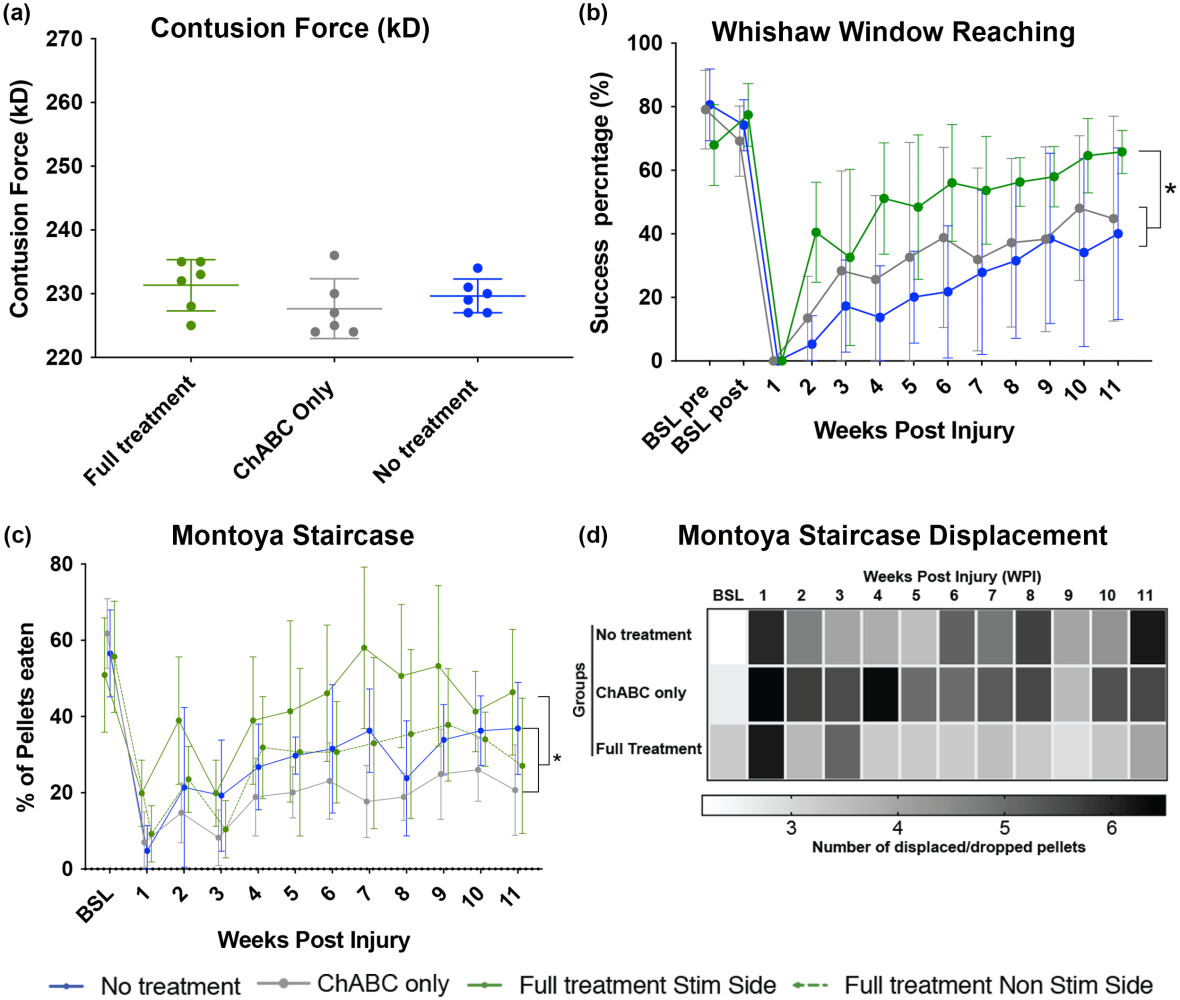
Increased muscle activity corresponds to recovery of skilled forelimb function. (a) no significant variation in the contusion force was observed, confirming consistent injuries between the groups (*F*_(2,15)_ = 1.342, *p* = .291, one‐way ANOVA). (b) Weekly behavioral assessments of reaching success rates in the Whishaw window reaching task indicate that the triple treatment group showed a significant increase in the number of sugar pellets retrieved, compared to the no treatment or LV‐ChABC only treatment groups (Time: *F*_(12,180)_ = 32.11, *p* < .0001; Treatment: *F*_(2,15)_ = 3.315, *p* = .064; Interaction: *F*_(24,180)_ = 1.712, *p* < .05 two‐way RM ANOVA, Tukey's *post‐hoc*). (c) Weekly behavioral assessments in the Montoya staircase task measuring the number of total pellets eaten indicated that the triple treatment group reached for the most pellets using the side contralateral to stimulation (Stim side) compared to the side ipsilateral to stimulation (Non‐stim side) (Time: *F*_(4.876,97.53)_ = 26.09, *p* < .0001; Treatment: *F*_(3,20)_ = 5.125, *p* < .01 Interaction: *F*_(33,220)_ = 1.705, *p* < .01, two‐way RM ANOVA, Tukey's *post‐hoc*) (d) Grayscale heat map indicating the degree of displacement and/or dropped pellets during the Montoya staircase task shows more accurate reach and grasp in the triple treatment group, compared to the no treatment and LV‐ChABC only groups (white = 0 pellets dropped/displaced; black = more than 6 pellets dropped/displaced). Error bars = Standard Deviation.

We first examined the contusion injury force (kD) measurements and confirmed that contusion injuries were consistent, with no significant differences in impact force measurements between treatment groups (*F*_(2,15)_ = 1.342, *p* = .291, one‐way ANOVA) (Figure 4a). To assess whether there was a significant increase in regain of upper limb skilled function, we scored all animals once per week in the Whishaw window reaching and the Montoya staircase tasks. A significant difference between the triple treatment group was observed when compared to the LV‐ChABC only and untreated groups in the Whishaw window reaching task (Figure 4b), with the triple treatment group showing a robust and consistent improvement in the percentage of successfully retrieved pellets throughout the testing period (Time: *F*_(12,180)_ = 32.11, *p* < .0001; Treatment: *F*_(2,15)_ = 3.315, *p* = .064; Interaction: *F*_(24,180)_ = 1.712, *p* = .026, two‐way RM ANOVA, Tukey's *post‐hoc*). For the Montoya staircase task, scores for the stimulated forelimb and the non‐stimulated forelimb were separately analyzed for the triple treatment group. This revealed a statistically significant improvement for the stimulated side of the triple treatment group, compared to the non‐stimulated side of the same group and the LV‐ChABC only and untreated groups (Time: *F*_(4.876,97.53)_ = 26.09, *p* < .0001; Treatment: *F*_(3,20)_ = 5.125, *p* < .01 Interaction: *F*_(33,220)_ = 1.705, *p* < .01 two‐way RM ANOVA, Tukey's *post‐hoc*) in the number of pellets retrieved (Figure 4c). By utilizing colored pellets we were also able to measure the number of displaced and/or dropped pellets per treatment group. The heat map representation demonstrates that the triple treatment group displaced the least number of pellets over the time course of 11 weeks when compared to the LV‐ChABC only and untreated groups (Figure 4d). These results reveal that the increased muscle activity we observed with chronic muscle recordings is associated with recovery of skilled upper limb motor function.

### 3.5 Terminal electrophysiology reveals increased muscle activity in the triple treatment combination group

**FIGURE 5:**
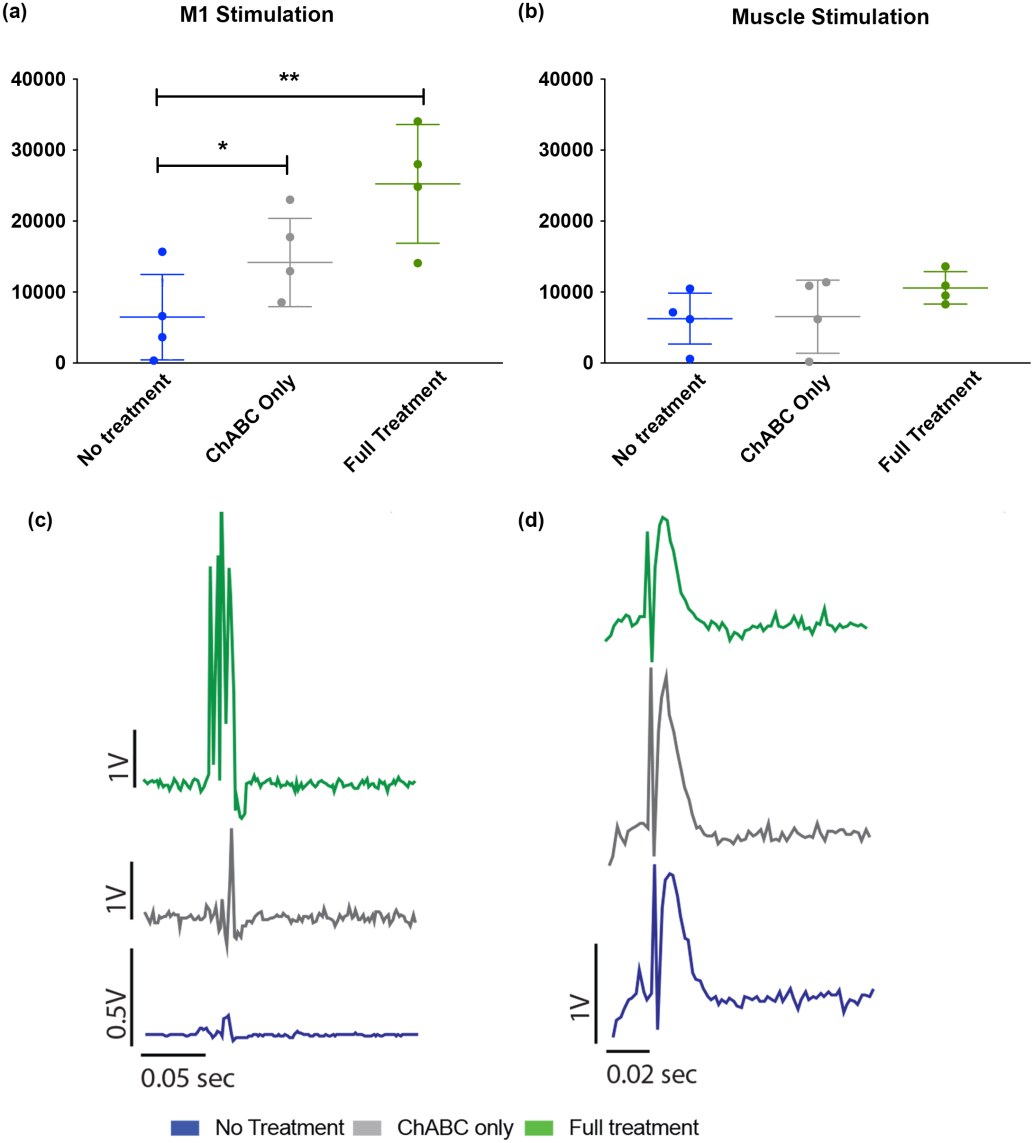
A triple combination treatment promotes increased evoked muscle activity during terminal cortical stimulation. Terminal electrophysiology performed 12 weeks after contusion injury under anesthesia. (a) Quantified area under the curve (AUC) (mV*mS) measurements from motor evoked potentials (MEPs) recorded from percutaneous EMG electrodes by stimulating the implanted M1 cortical epidural electrode. The triple treatment group and the LV‐ChABC only treatment group showed a significant increase in muscle activity compared to the no treatment group (*F*_(2,12)_ = 9.288, *p* = .004 one‐way ANOVA, Tukey's *post‐hoc*). (b) Quantified AUC (mV*mS) measurements from MEPs recorded from percutaneous EMG electrodes by stimulating the implanted EMG electrode through the implanted brain interface board. No differences in muscle activity were observed between the treatment groups when the muscle was being stimulated (*F*_(2,12)_ = 1.551, *p* = .252 one‐way ANOVA). (c) Colored representative rectified average traces (10 triggers/average) of MEPs from the different groups, obtained by stimulating the M1. The no treatment group has a different *y*‐axis scale bar to achieve better trace representation. (d) Colored representative rectified average traces (10 triggers average) of MEPs from the different groups, obtained by peripheral muscle stimulation.

Chronic muscle recordings at the terminal time point (12 weeks post‐injury) demonstrated that when stimulating the motor cortex under anesthesia, the triple combination treatment leads to a significant increase in muscle activity, when compared to the LV‐ChABC only and the untreated group, quantified by AUC measurements (*F*_(2,12)_ = 9.288, *p* < .01 one‐way ANOVA, Tukey's *post‐hoc*) (Figure 5a,c). To confirm that the increased MEP AUC response was not due to muscle atrophy or trauma caused by the implanted electrodes, we stimulated the bicep muscles through the recording interface while recording from percutaneous EMG wires, which revealed that there was no significant difference between the AUC recorded from the different groups (*F*_(2,12)_ = 1.551, *p* = .251, one‐way ANOVA) (Figure 5b,d). Therefore, the lasting increase in muscle activity observed during awake recordings can also be observed under anesthesia, where the corticospinal tract (CST) is not already primed by voluntary awake movements. Moreover, these data suggest that the increase in muscle activity is not due to any physical damage in the muscles such as atrophy or trauma from the electrodes but is likely due to the triple treatment combination administered.

### 3.6 Histological assessments of lesion spread and CSPG degradation

EC staining of serial sections spanning the cervical contusion injury revealed extensive tissue damage at the injury epicenter (spinal level C7), with almost complete destruction of spinal gray matter and only the borders of the white matter left intact (Figure S1). Analysis of the rostro‐caudal extent of injury showed the injury extended into spinal levels C6 and C8, although at these levels the damage was located mainly in dorsal white matter regions (Figure S1). C‐4‐S immunohistochemistry was also performed in serial sections spanning the injury, to determine effectiveness of LV‐ChABC treatment. Positive immunostaining for C‐4‐S was apparent as diffuse staining across spinal cord sections from animals that were treated with LV‐ChABC as a single treatment, or with the full combination treatment, while no positive C‐4‐S staining was observed in spinal cords from the untreated group (Figure S1).

### 3.7 Biceps motor neuron pool mapping

**FIGURE 6:**
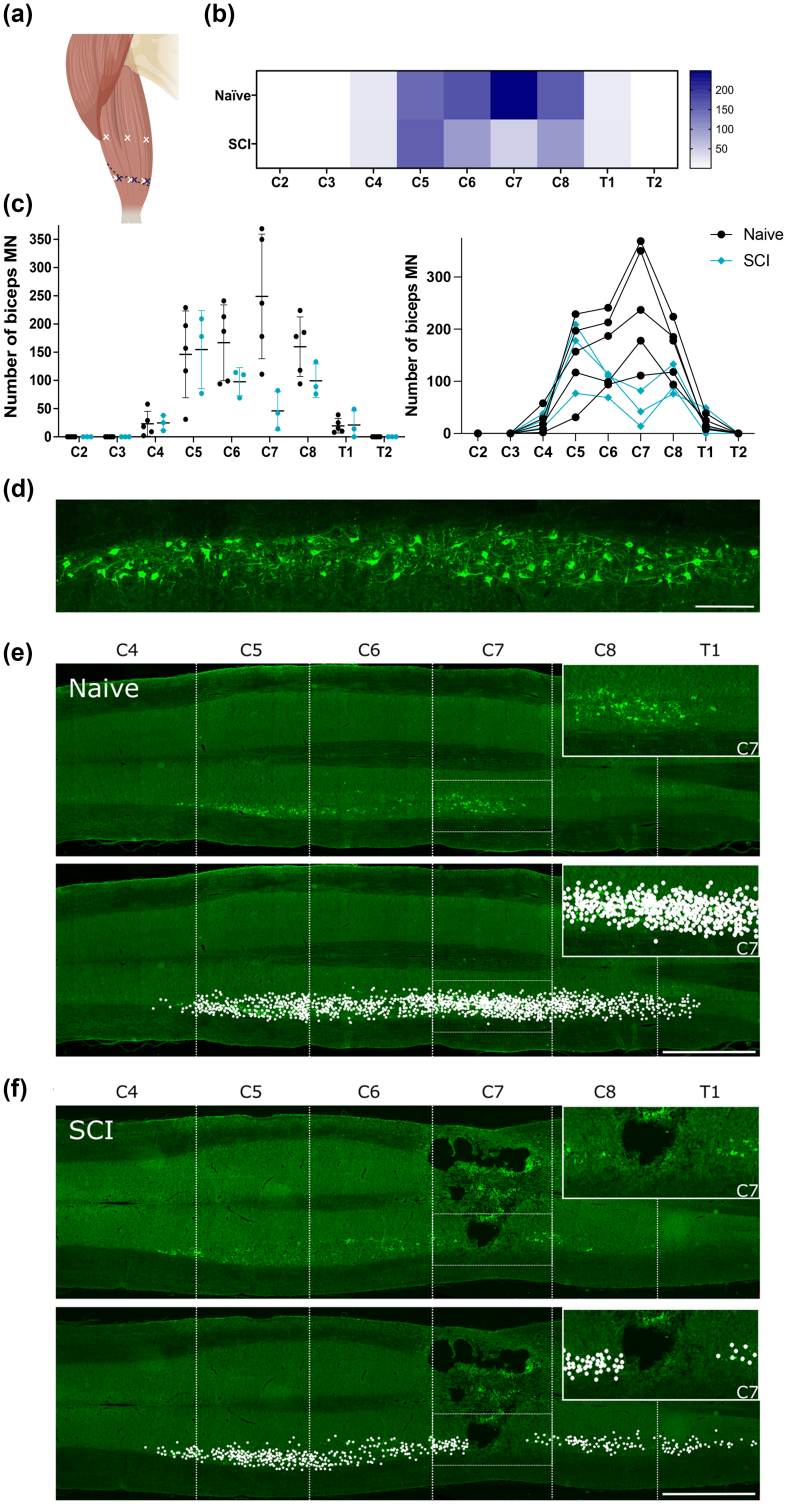
*Biceps brachii* spinal motor neuronal pool mapping. (a) Schematic showing deep (white) and superficial (blue) injection sites in the two heads of the right biceps. The short head of the biceps received two deep and two superficial injections aiming for the motor endplates (dashed line) and two deep injections aiming at the region halfway between the proximal end of the muscle and the motor endplates. The long head of the biceps received one deep and one superficial injection aiming for the motor endplates and one deep injection aiming at the region halfway between the proximal end of the muscle and the motor endplates. (b) Heat map graph showing distribution of biceps motor neurons in the naive and SCI groups. Biceps motor neurons were found between spinal levels C4 to T1, with highest concentration in C7. After spinal cord injury the numbers of biceps motor neurons were dramatically reduced in C7 (lesion epicenter) and slightly decreased in adjacent levels C6 and C8. (c) Bar and line graphs showing the number of biceps motor neurons counted at each spinal levels for the naïve and SCI groups. Individual animals were plotted as symbols in the bar graph and individual lines in the line graph. After SCI, the number of motor neurons in the lesion epicenter (C7) is reduced compared to naive. (d) CTB‐positive biceps motor neurons. Scale bar = 250 μm. (e,f) Horizontal sections of the spinal ventral horn of a naïve (e) and a SCI (f) rat that had the right biceps injected with CTB. C7 (lesion epicenter) is showed in detail at the top right of each image. The bottom image on each panel shows a 2D reconstruction of all counted CTB positive neurons (white dots) present on all analyzed slides of a representative animal. Biceps motor neurons were present from C4 to T1, more densely between C5 and C8. SCI reduced the amount of motor neurons present in the lesion epicenter (C7) and neighboring levels. Scale bar = 2 mm. Error bars = Standard Deviation.

We next performed motor neuron pool mapping studies to determine the extent to which biceps motor neurons were affected by our C7 contusion injury, since previous studies have shown wide variability in the reported segmental distribution of biceps motor neurons (Bacskai et al., 2013). CTB was injected into the *biceps brachii* (Figure 6a) to map the distribution of biceps motor neurons over spinal segments C2–T2 in naïve (uninjured) rats and after C7 contusion injury (Figure 6). In naive animals, CTB‐positive biceps motor neurons (Figure 6d) were identified across spinal levels C4 to T1, with the highest concentration at C7 and lowest concentration at C4 and T1 (Table 1, Figure 6b,c,e). No biceps motor neurons were found at spinal levels C2, C3, and T2 in any of the animals studied (Table 1, Figure 6b,c). Naive and SCI rats had similar numbers of biceps motor neurons at levels C4 (naive: 23.2 ± 21.9, SCI: 24.67 ± 13.5; average ± SD, *p* = .7857), C5 (naive: 146.2 ± 76.9, SCI: 154.67 ± 69; average ± SD, *p* = .999), and T1 (naive: 19.4 ± 12.8, SCI: 21 ± 25.2; average ± SD, *p* = .999). Biceps motor neuron numbers in SCI animals were reduced after SCI at levels C6 (naive: 166.8 ± 67, SCI: 97.67 ± 24.9; average ± SD, *p* = .3928) and C8 (naive: 159.8 ± 52.8, SCI: 99.33 ± 29.9; average ± SD, *p* = .1428), compared to the naïve animals, although the difference was not significant. At the lesion epicenter, spinal level C7, the SCI group had a significant reduction of biceps motor neuron numbers compared to the naive group (naive: 249 ± 110.5, SCI: 46 ± 34.2; average ± SD, *p < .05). Thus, the C7 contusion injury affects the largest pool of biceps motor neurons located at spinal level C7, leaving some biceps motor neuron pools intact above and below the injury level.

**TABLE 1:** Number and distribution of CTB‐labeled MNs following *biceps brachii* muscle injection

	C2	C3	C4	C5	C6	C7	C8	T1	T2	Total
Naïve #1	0	0	29	197	213	350	178	10	0	977
Naïve #2	0	0	9	229	241	369	224	39	0	1111
Naïve #3	0	0	58	157	187	237	185	8	0	832
Naïve #4	0	0	18	117	99	178	94	25	0	531
Naïve #5	0	0	2	31	94	111	118	15	0	371
Average Naïve	0	0	23	146	167	249	160	19	0	764
SCI #1	0	0	11	77	69	14	89	0	0	260
SCI #2	0	0	38	209	110	82	133	14	0	586
SCI #3	0	0	25	178	114	42	76	49	0	484
Average SCI	0	0	25	155	98	46	99	21	0	443

## 4 DISCUSSION

Here, we utilized a novel approach of chronic longitudinal EMG recordings from intramuscularly implanted electrodes as an experimental tool for assessing muscle activity and regain of function after a cervical contusion injury in adult rats. Intra‐cortically induced MEPs have been previously used as an assessment method, and have been applied at a single terminal time point to assess conduction after injury (Fouad et al., 2001; Hall & Lindholm, 1974; Jack et al., 2018). However, in the present study epidurally induced MEPs have been longitudinally followed in the same animals from pre‐injury through to a chronic injury state over a 12 week period. While standardized behavioral (Basso et al., 1995; Gale et al., 1985; James et al., 2015; Montoya et al., 1991; Whishaw & Pellis, 1990) and neuroanatomical (Anderson et al., 2005, 2007) assessments can provide valuable information for assessing functional outcome following SCI and treatment interventions, chronic EMG recordings measured in awake, freely moving animals may provide an additional outcome measure for accurately assessing muscle function as an index of motor recovery. Using this methodology our data revealed that a novel triple combination of treatments (the neuroplasticity‐promoting agent ChABC, plus long‐term epidural cortical stimulation, plus skilled reaching rehabilitation) resulted in a significant increase in bicep muscle activity, both when evoked through stimulation and when exerted voluntarily. Increased muscle activity was associated with improved upper limb function, assessed by skilled reach and grasp behavioral tasks. Finally, increased MEP recordings from terminal electrophysiology experiments under anesthesia showed similar increased muscle activity in the triple treatment group, supporting our observations that this novel combination therapy promotes recovery of upper limb function after cervical contusion injury.

### 4.1 Neuroplasticity‐inducing agents applied as combinatorial therapies

It has previously been established that the injured CNS has the ability for some spontaneous recovery after injury (Bareyre et al., 2004; Raineteau et al., 2001; Rosenzweig et al., 2010; Steeves et al., 2011). This ability has been boosted and modified through the use of physical rehabilitation and neuroplasticity‐enhancing agents in previous studies. For example, neuroplasticity‐inducing agents such as anti‐Nogo therapy and ChABC can lead to extensive functional recovery after CNS injury when combined with physical rehabilitation and training (Chen et al., 2017; García‐Alías et al., 2011, 2012; Wahl & Schwab, 2014; Wang et al., 2011). Here, we are the first to examine the effects of combining the neuroplasticity‐inducing agent LV‐ChABC and physical rehabilitation in conjunction with epidural electrical stimulation of the motor cortex in a clinically relevant cervical contusion injury model. Surprisingly, in the current study the LV‐ChABC single treatment group showed limited improvement in regain of skilled function. This is in contrast to a number of previous studies where treatment with the bacterial ChABC enzyme has enabled recovery of motor function after SCI as a single treatment (Bradbury et al., 2002; Hu et al., 2018; Jefferson et al., 2011; Warren et al., 2018). However, there is increasing evidence that the effects of ChABC are more potent when combined with an additional therapeutic, such as cellular or nerve grafts, myelin inhibitors, biomaterials, and neurotrophic factors (Alilain et al., 2011; Fouad et al., 2005; Führmann et al., 2018; Houle et al., 2006; Kanno et al., 2014; Karimi‐Abdolrezaee et al., 2010; Zhao & Fawcett, 2013). Furthermore, when applied to more clinically relevant contusion or compression models of SCI, beneficial effects of ChABC have been more limited (Tom et al., 2009), unless given as a multiple therapy (Suzuki et al., 2017) or as a gene therapy (Bartus et al., 2014; Burnside et al., 2018; James et al., 2015). Lentiviral gene delivery of ChABC is thought to be more effective than ChABC bacterial enzyme delivery since it more potently digests CSPGs in the injured spinal cord, which has multiple effects including modulating the immune response (Bartus et al., 2014; Didangelos et al., 2014) and enhancing plasticity of descending systems (Burnside et al., 2018), targeting two main goals for treating SCI (Hryciw et al., 2018). In the current study, we were unable to detect recovery of skilled forelimb function following LV‐ChABC treatment alone. There are several potential reasons. First, the current study used a viral vector one order of magnitude lower in titer than previous work. It is not easy to directly compare the current delivery potency with our dual vector immune‐evasive system (Burnside et al., 2018), but we observed effective CSPG digestion at the final time point over several spinal levels spanning the lesion in both the single and combination groups, suggesting effective ChABC delivery. A more likely possibility is the behavioral outcome measurements. In a previous study, using Whishaw reaching apparatus, we have found first time “hits” (successful pellet retrievals taking one attempt) to be more sensitive than “successful reaches” in detecting improvements in reach and grasp (Burnside et al., 2018). It may be that this more sensitive measure could have detected improvements following LV‐ChABC alone. The analysis of “successful reaches” may perhaps be itself more sensitive to observed improvements in bicep function elicited only in the combination group, improving gross movement of the paw toward the pellet. Similarly, despite the increase in bicep activity, the actual grip strength was not improved by the full treatment, suggesting the rehabilitation/stimulation effect is greater in proximal arm muscles. Furthermore, daily rehabilitation with the Montoya staircase task may have skewed our capacity to detect task‐specific improvements in the Whishaw task and vice versa (García‐Alías et al., 2009). Despite the lack of robust effects with LV‐ChABC as a single treatment, a clear trend for improvement was observed in the LV‐ChABC single treatment group in several assessments, for example motor evoked potential duration, recovery during the first 6 weeks of Whishaw reaching, and significantly increased muscle activity in terminal MEP recordings, compared to the untreated group. However, as these effects were modest, the addition of training and stimulation was likely required to engage and strengthen the correct circuitry to enable significant functional improvement. Further investigation is required to fully determine the reasons for lack of effect of the single treatment, or whether we can stratify and evaluate responders and nonresponders, as in previous work targeting CSPG signaling (Lang et al., 2015). However, it is likely that to achieve optimal functional recovery with a ChABC enzyme, or a ChABC vector especially if a less potent variant than current gold‐standard vectors (Muir et al., 2019) requires training and/or activity as an adjunct, as has been suggested for most neuroplasticity‐promoting therapies (Torres‐Espín et al., 2018) and successfully applied here.

### 4.2 Potential mechanisms underlying recovery of function after combination therapy

In order to interpret the biceps muscle function recovery data, we performed motor neuron mapping studies. Since previous studies have shown inconsistencies regarding their distribution across C3–C5 (Tosolini & Morris, 2012), C4–C5 (McKenna et al., 2000; Ryan et al., 1998), C5–C6 (Bennett et al., 1983), C5–C7 (Gramsbergen et al., 1996) or over a longer distribution, spanning C4–C7 (Bacskai et al., 2013; Bertelli et al., 1995), or C4–C8 (Tada et al., 1979) we thought it was essential to determine the segmental distribution in the rat strain and injury model used in the current study. Our results are in agreement with the latter studies, showing biceps motor neurons extending from C4 to C8, with the largest pool at C7. Thus, our C7 contusion caused significant damage to biceps motor neurons. Thus, the effects of the combination treatment on restoring biceps muscle function are likely to be due to targeting both damaged muscle circuitry as well as spared circuitry immediately rostral to the injury site, similar to previous studies (Nakamura et al., 1997).

While anatomical tract tracing of descending motor pathways was not assessed in the present study, it is likely that the combination treatment also elicited some changes in descending spinal motor circuitry. For example, ChABC treatment alone can enable increased CST reinnervation within the denervated spinal cord in models of SCI (Starkey et al., 2012) and stroke (Soleman et al., 2012). High intensity upper limb rehabilitative training can elicit enhanced CST plasticity (Fenrich et al., 2021) and cortical electrical stimulation can elicit axonal regeneration and sprouting of descending motor pathways after SCI (Carmel et al., 2010; Carmel & Martin, 2014; Hamid et al., 2008; Jack et al., 2020; Zareen et al., 2017). Moreover, a recent study suggested the reactivation of the growth‐promoting Jak/Stat developmental pathways as a potential mechanism activated by cortical electrical stimulation (Zareen et al., 2018). Similar evidence has been reported in peripheral nerve injury studies which revealed that electrical stimulation can promote axonal regeneration and sprouting (Ahlborn et al., 2007; Asensio‐Pinilla et al., 2009).

### 4.3 Cortical epidural electrical stimulation as an adjunct therapy for SCI

The current study represents the first attempt to harvest beneficial stimulation‐induced axonal sprouting and excitability (Field‐Fote, 2015) in a permissive environment created by the neuroplasticity‐enhancing effects of LV‐ChABC and combine these with extensive physical rehabilitation that can consolidate and strengthen meaningful connectivity in the injured spinal cord (Edgerton et al., 2001). Furthermore, we applied this combinational paradigm in a clinically relevant cervical contusion injury model. This is important since the majority of human SCIs occur at the cervical level, affecting the upper extremities, and the highest priority for regaining independence and improved quality of life for these individuals is to regain hand function (Anderson, 2004; Singh et al., 2014). Previous studies have shown that cortical epidural stimulation can have favorable outcomes in a unilateral pyramidotomy injury model leading to axonal sprouting (Carmel et al., 2010, 2013; Carmel & Martin, 2014). A recent study from the same group also utilized intermittent theta burst (iTBS) cortical epidural stimulation in conjunction with targeted transpinal direct current stimulation (tsDCS) as a dual treatment repair strategy for a moderate cervical contusion injury in rats and reported regain of locomotion and forepaw manipulation along with stimulation enhanced axonal sprouting rostral and caudal to injury (Zareen et al., 2017). Furthermore, evidence from stroke‐related and cortical lesion studies (Adkins et al., 2008), both experimental and clinical (Levy et al., 2008), suggests that cortical stimulation can increase skilled motor function when used as a treatment in parallel with rehabilitation (Adkins‐Muir & Jones, 2013; Kleim et al., 2003; Teskey et al., 2003). This has also recently been applied to SCI, where a single session of acute cortical electrical stimulation was shown to enhance the efficacy of rehabilitative training after cervical SCI (Batty et al., 2020). Therefore, there is compelling evidence supporting the notion that cortical electrical stimulation can be used to restore skilled functional motor outcomes, and effects can be enhanced with training and plasticity‐promoting therapies.

### 4.4 Timing of combination therapies

An important question related to combinational therapeutic approaches is the optimal timing for which each therapy should be administered. Several studies have investigated the importance of the timing of treatment administration (García‐Alías et al., 2011; Gonzenbach et al., 2011; van den Brand et al., 2012), and have similarly concluded that for the optimal rehabilitation paradigm to be created, the timing of administering each intervention should be aligned with the already occurring spontaneous recovery processes, therefore utilizing the given “window of opportunity” (García‐Alías et al., 2009). In the present study, the administration of LV‐ChABC occurred acutely, while the other components of the triple treatment (daily cortical stimulation and skilled reach training) were initiated after a delay of 1 week post‐injury. This is in line with anti‐Nogo studies for SCI, where early anti‐Nogo administration (either immediate or within 1 week post‐injury) is found to be optimal, after which the neuroplasticity‐promoting effects are diminished (Gonzenbach et al., 2011), and the rehabilitation component should be sequential, rather than concurrent (Chen et al., 2017). Previous studies have also demonstrated that a delayed administration of rehabilitation resulted in a significant reduction in functional recovery and limited improvement of motor functions (Norrie et al., 2005; Torres‐Espín et al., 2018), thus providing evidence supporting early rehabilitation intervention. However, there is still relatively little known about optimal timing of combinatorial therapies and further work should focus on determining critical time windows of neuroplasticity treatments in combination with training and stimulation paradigms. Finally, a limitation of the study is the use of only female rats. While this was justified (based on lower attrition and better long‐term welfare with single housing), sex differences should be further examined in future studies.

Applying multiple therapeutic interventions in combination may be the most optimal approach for achieving meaningful functional recovery following traumatic SCI. Here our novel combination of a neuroplasticity‐promoting agent, together with long‐term repetitive cortical epidural stimulation and rehabilitative training resulted in increased muscle activity and recovery of skilled upper limb function after cervical contusion injury. Thus, our triple therapeutic approach along with our novel assessment methodology represent a promising advance and supports the further development of combination treatments for enabling regain of upper limb and hand function after traumatic SCI.

## DECLARATION OF TRANSPARENCY

The authors, reviewers and editors affirm that in accordance to the policies set by the *Journal of Neuroscience Research*, this manuscript presents an accurate and transparent account of the study being reported and that all critical details describing the methods and results are present.

## AUTHOR CONTRIBUTIONS

All authors had full access to all the data in the study and take responsibility for the integrity of the data and the accuracy of the data analysis. *Conceptualization*, E.J.B., S.B.M., and E.S.; *Methodology*, E.S.; *Investigation*, E.S., A.S., N.R., K.B., and E.R.B.; *Software*, E.S.; *Formal Analysis*, E.S.; *Resources*, F.D.W.; *Writing – Original Draft*, E.S.; *Writing – Review & Editing*, E.J.B. and E.S.; *Visualization*, E.S.; *Supervision*, E.J.B. and S.B.M.; *Funding Acquisition*, E.J.B.

## CONFLICT OF INTEREST

The authors declare no competing financial interests.

### PEER REVIEW

The peer review history for this article is available at https://publons.com/publon/10.1002/jnr.25111.

## Supporting information


**FIGURE S1** Establishing chronic muscle recordings through a feasibility study. (a) Maximum voluntary contraction (MVC) measurements from awake animals at 11 weeks post‐injury. The triple treatment group showed a significant increase in MVC compared to the no treatment and the stimulation only group (*F*_(3,12)_ = 12.86, *p* < .001 one‐way ANOVA, Tukey's *post‐hoc*). Average of three trials. (b) Muscle recoding traces during MVC recordings. Colored boxes below the traces indicate the group and response duration and black boxes indicate the timestamped start of each trial. (c) Grip strength apparatus force measurements at 11 weeks post‐injury where no difference was observed between the force values obtained from the different groups (*F*_(3,12)_ = 1.700, *p* < .089 one‐way ANOVA). Error bars = Standard Deviation.
**FIGURE S2** Extent of tissue damage after bilateral C7 spinal contusion injury. Eriochrome cyanine (EC)‐stained transverse sections of the spinal cord spanning the injury epicenter and extending rostro‐caudally, and respective spinal level atlas schematics (Paxinos). Tissue damage to the dorsal white matter extends from C6 to C8 spinal levels. The epicenter of injury was in C7, where only the border of the white matter is preserved. Scale bar = 500 μm
**FIGURE S3** Chondroitin‐4‐sulfate (C‐4‐S) staining to show cleavage of matrix components after intraspinal injection of the chondroitinase vector. Representative examples of C‐4‐S staining in transverse spinal cord sections from each treatment group. Lack of C‐4‐S immunoreactivity confirmed no CSPG degradation in the no treatment group rostral to the injury site (+60 μm; yellow line outlining section) and at the injury epicenter. In contrast, positive C‐4‐S immunostaining was apparent throughout the spinal cord in sections rostral to the injury site (+60 μm) and at the injury epicenter for both the ChABC‐only treatment group and the triple treatment group. Scale bar: 500 μm

Transparent Science Questionnaire for Authors

## Data Availability

The data that support the findings of this study are available on request from the corresponding author. The data are not publicly available due to privacy or ethical restrictions.
