# Supplementary material for: Chronic muscle recordings reveal recovery of forelimb function in spinal injured female rats after cortical epidural stimulation combined with rehabilitation and chondroitinase ABC
Source: J Neurosci Res. 2022 Aug 2;100(11):2055–76. doi: 10.1002/jnr.25111 (PMC9544922; doi:10.1002/jnr.25111)
Supplement: Supplementary file 1 — FIGURE S1 Establishing chronic muscle recordings through a feasibility study. (a) Maximum voluntary contraction (MVC) measurements from awake animals at 11 weeks post‐injury. The triple treatment group showed a significant increase in MVC compared to the no treatment and the stimulation only group (F (3,12) = 12.86, p < .001 one‐way ANOVA, Tukey's post‐hoc). Average of three trials. (b) Muscle recoding traces during MVC recordings. Colored boxes below the traces indicate the group and response duration and black boxes indicate the timestamped start of each trial. (c) Grip strength apparatus force measurements at 11 weeks post‐injury where no difference was observed between the force values obtained from the different groups (F (3,12) = 1.700, p < .089 one‐way ANOVA). Error bars = Standard Deviation. FIGURE S2 Extent of tissue damage after bilateral C7 spinal contusion injury. Eriochrome cyanine (EC)‐stained transverse sections of the spinal cord spanning the injury epicenter and extending rostro‐caudally, and respective spinal level atlas schematics (Paxinos). Tissue damage to the dorsal white matter extends from C6 to C8 spinal levels. The epicenter of injury was in C7, where only the border of the white matter is preserved. Scale bar = 500 μm FIGURE S3 Chondroitin‐4‐sulfate (C‐4‐S) staining to show cleavage of matrix components after intraspinal injection of the chondroitinase vector. Representative examples of C‐4‐S staining in transverse spinal cord sections from each treatment group. Lack of C‐4‐S immunoreactivity confirmed no CSPG degradation in the no treatment group rostral to the injury site (+60 μm; yellow line outlining section) and at the injury epicenter. In contrast, positive C‐4‐S immunostaining was apparent throughout the spinal cord in sections rostral to the injury site (+60 μm) and at the injury epicenter for both the ChABC‐only treatment group and the triple treatment group. Scale bar: 500 μm [file JNR-100-2055-s002.docx]

**SUPPLAMENTARY MATERIAL**


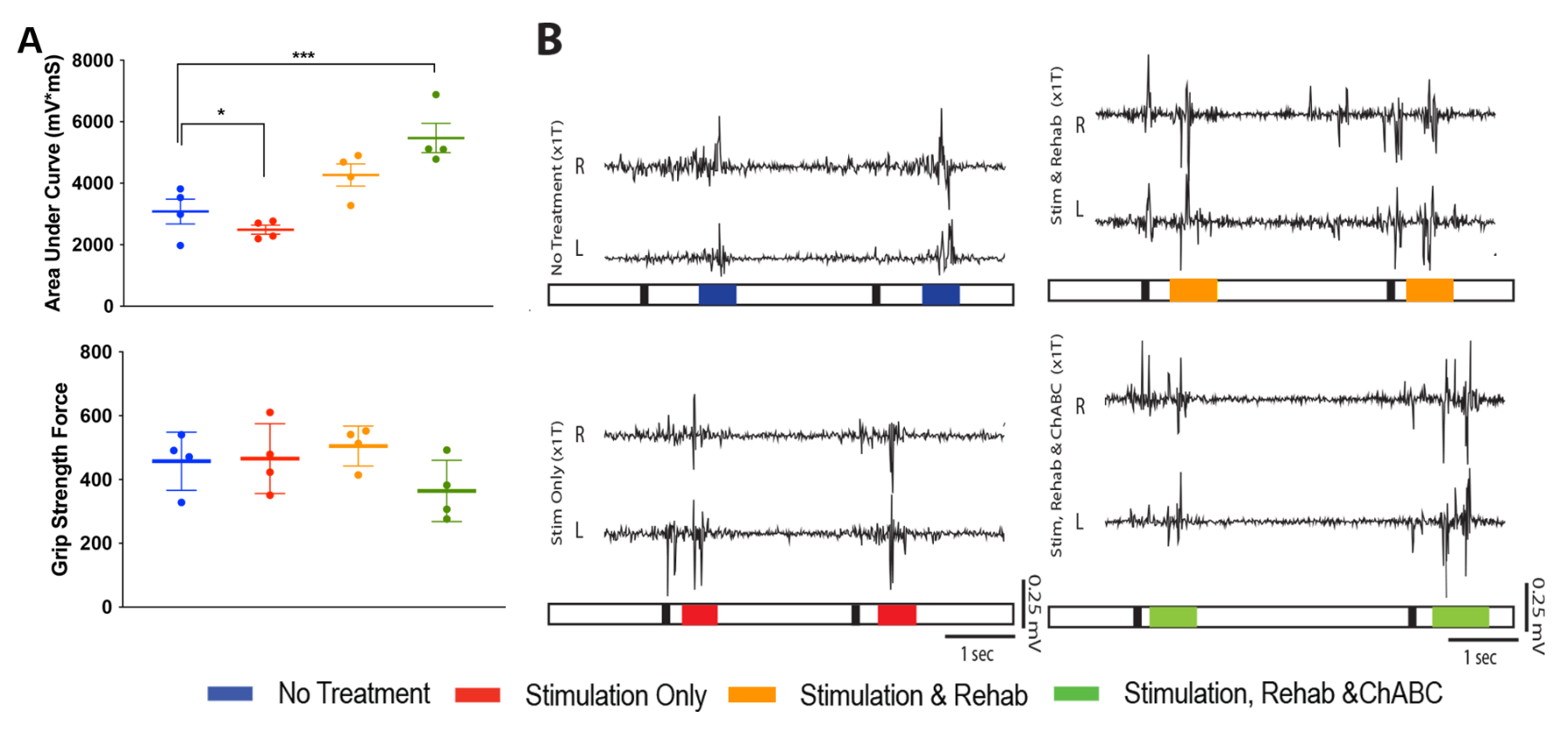


**Supplementary Figure 1.** Establishing chronic muscle recordings through a feasibility study. (**A**) Maximum voluntary contraction (MVC) measurements from awake animals at 11 weeks post injury. The triple treatment group showed a significant increase in MVC compared to the no treatment and the stimulation only group (F_3,12_=12.86, p<0.001 one-way ANOVA, Tukey’s *post-hoc*). Average of 3 trials. **(B)** Muscle recoding traces during MVC recordings. Colored boxes below the traces indicate the group and response duration and black boxes indicate the timestamped start of each trial. (**C**) Grip strength apparatus force measurements at 11 weeks post injury where no difference was observed between the force values obtained from the different groups (F_3,12_= 1.700, p<0.089 one-way ANOVA)


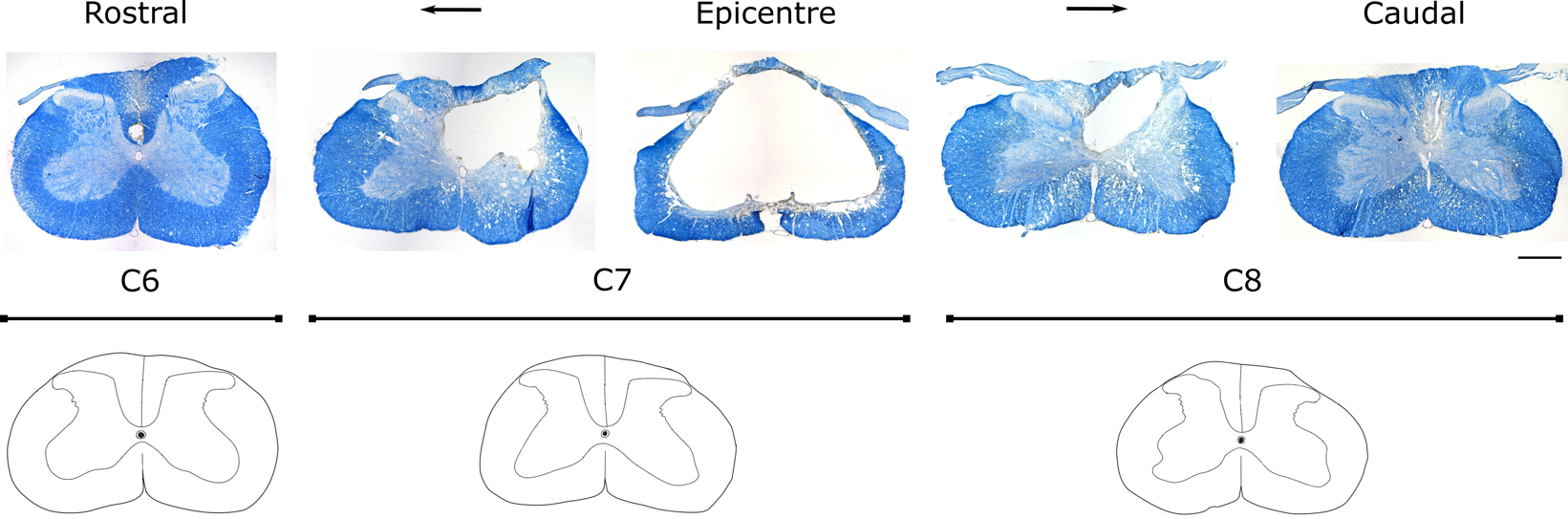


**Supplementary Figure 2.** Extent of tissue damage after bilateral C7 spinal contusion injury. Eriochrome cyanine (EC)-stained transverse sections of the spinal cord spanning the injury epicentre and extending rostro-caudally, and respective spinal level atlas schematics (Paxinos). Tissue damage to the dorsal white matter extends from C6 to C8 spinal levels. The epicentre of injury was in C7, where only the border of the white matter is preserved. Scale bar = 500um


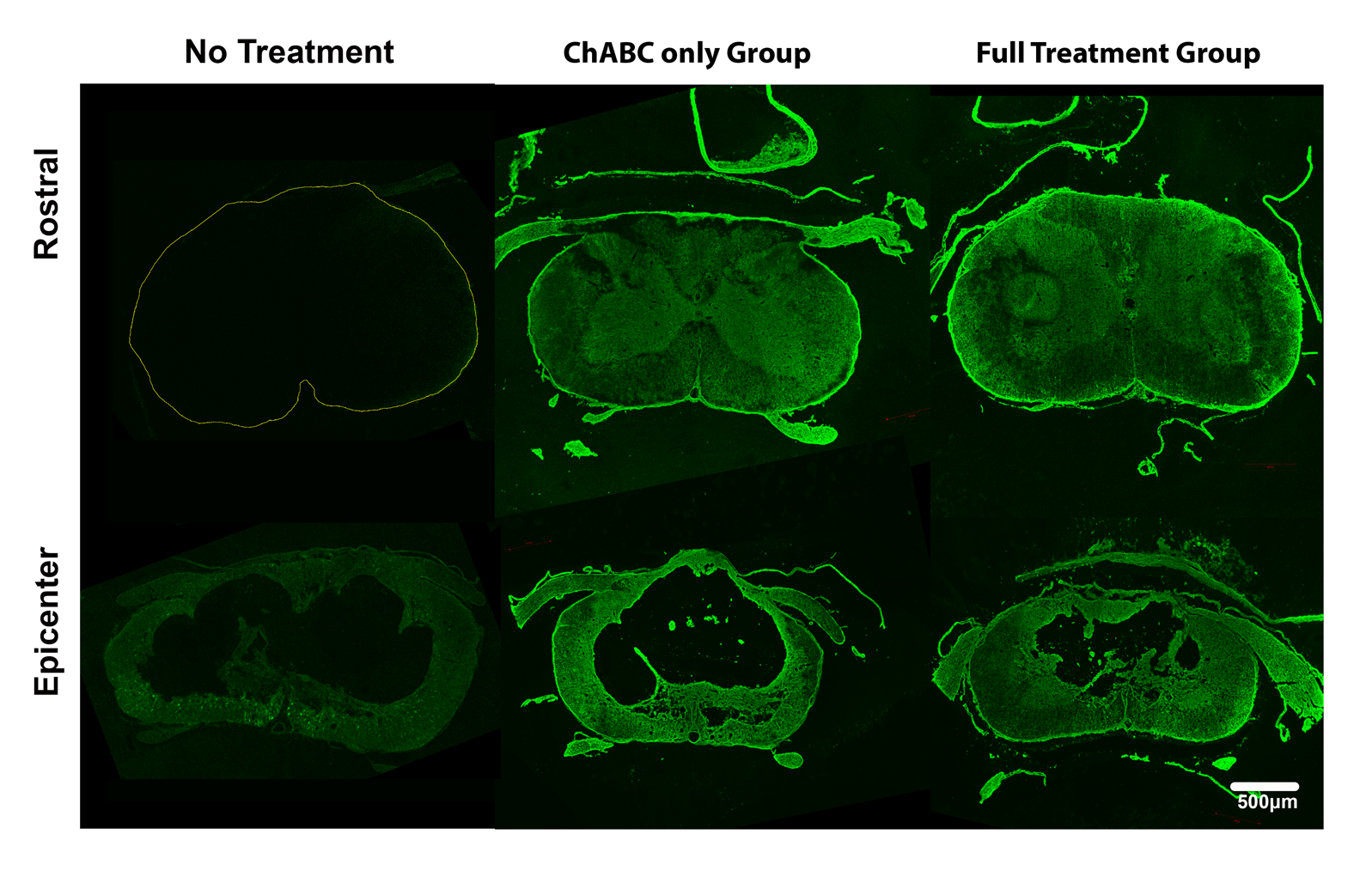


**Supplementary Figure 3.** Chondroitin-4-sulfate (C-4-S) staining to show cleavage of matrix components after intraspinal injection of the chondroitinase vector. Representative examples of C-4-S staining in transverse spinal cord sections from each treatment group. Lack of C-4-S immunoreactivity confirmed no CSPG degradation in the no treatment group rostral to the injury site (+60μm; yellow line outlining section) and at the injury epicentre. In contrast, positive C-4-S immunostaining was apparent throughout the spinal cord in sections rostral to the injury site (+60μm) and at the injury epicentre for both the ChABC-only treatment group and the triple treatment group. Scale bar: 500μm.
